# Side-by-side comparison of published small molecule inhibitors against thapsigargin-induced store-operated Ca^2+^ entry in HEK293 cells

**DOI:** 10.1371/journal.pone.0296065

**Published:** 2024-01-23

**Authors:** Katherine Norman, Karen E. Hemmings, Heba Shawer, Hollie L. Appleby, Alan J. Burnett, Nurasyikin Hamzah, Rajendra Gosain, Emily M. Woodhouse, David J. Beech, Richard Foster, Marc A. Bailey

**Affiliations:** 1 School of Chemistry, University of Leeds, Leeds, West Yorkshire, United Kingdom; 2 Discovery and Translational Science Department, Leeds Institute of Cardiovascular and Metabolic Medicine, School of Medicine, University of Leeds, Leeds, West Yorkshire, United Kingdom; Indiana University School of Medicine, UNITED STATES

## Abstract

Calcium (Ca^2+^) is a key second messenger in eukaryotes, with store-operated Ca^2+^ entry (SOCE) being the main source of Ca^2+^ influx into non-excitable cells. ORAI1 is a highly Ca^2+^-selective plasma membrane channel that encodes SOCE. It is ubiquitously expressed in mammals and has been implicated in numerous diseases, including cardiovascular disease and cancer. A number of small molecules have been identified as inhibitors of SOCE with a variety of potential therapeutic uses proposed and validated *in vitro* and *in vivo*. These encompass both nonselective Ca^2+^ channel inhibitors and targeted selective inhibitors of SOCE. Inhibition of SOCE can be quantified both directly and indirectly with a variety of assay setups, making an accurate comparison of the activity of different SOCE inhibitors challenging. We have used a fluorescence based Ca^2+^ addback assay in native HEK293 cells to generate dose-response data for many published SOCE inhibitors. We were able to directly compare potency. Most compounds were validated with only minor and expected variations in potency, but some were not. This could be due to differences in assay setup relating to the mechanism of action of the inhibitors and highlights the value of a singular approach to compare these compounds, as well as the general need for biorthogonal validation of novel bioactive compounds. The compounds observed to be the most potent against SOCE in our study were: 7-azaindole 14d (12), JPIII (17), Synta-66 (6), Pyr 3 (5), GSK5503A (8), CM4620 (14) and RO2959 (7). These represent the most promising candidates for future development of SOCE inhibitors for therapeutic use.

## Introduction

The calcium (Ca^2+^) ion is involved in key cellular processes including signalling, mitochondrial regulation, motility and apoptosis. It is typically found at ~100 nM intracellular free concentration and ~2 mM extracellularly, a ~20,000× difference [1]. The main source of Ca^2+^ influx into non-excitable cells is store-operated (or capacitative) Ca^2+^ entry (SOCE), in which eukaryotic cells store Ca^2+^ in the endoplasmic reticulum (ER), using sarco-/endoplasmic reticulum Ca^2+^ ATPase (SERCA) pumps to counteract the continual leakage back into the cytoplasm. If the SERCA pump is blocked, or during a signalling event, the ER Ca^2+^ stores will deplete, and SOCE is activated. This model of Ca^2+^ influx was first proposed in 1986 [2], and the resulting current, known as the Ca^2+^-release activated Ca^2+^ current (I_CRAC_), was identified in 1997 [3]. ORAI1 is the pore-forming plasma membrane (PM) protein subunit of the CRAC channel encoding SOCE. It was identified in 2006 [4–6] and has three mammalian homologs, ORAI1, ORAI2, and ORAI3. They share no close homology with any known proteins [7]. The ER membrane (EM) protein stromal interaction molecule 1 (STIM1) was identified in 2005 as a transmembrane ER Ca^2+^-sensor [8]. STIM1 units cluster forming punctae with ORAI1 opening the channel pore allowing Ca^2+^ influx into the cytosol allowing ER store refilling [9]. The X-ray crystal structure of *Drosophila melanogaster* ORAI1, (73% sequence homology with humans) was solved in 2012 to a 3.35 Å resolution [10] revealing four transmembrane domains and a hexamer about the pore and supported by recent functional and cryo-EM structural data [11–13].

Loss-of-function mutations in ORAI1 and STIM1 are associated with immunodeficiency [14–16], autoimmunity [17], muscle hypotonia [18,19] and dental enamel defects [20]. Whilst gain-of-function mutations have been linked to platelet disorders [21] and myopathy [22]. Much research has focused on ORAI1 and SOCE as a drug target in immunology and inflammatory disease [23–26]. However, a growing body of evidence links SOCE/ORAI1 to cardiovascular and cardiorespiratory pathologies [27–30]. Small molecule inhibition of SOCE and genetic disruption of *Orai1* using a dominant negative mutant (in cardiac myocytes) was cardioprotective following pressure overload heart failure induction [31]. Both ORAI1 and ORAI2 are upregulated in pulmonary arterial smooth muscle cells (PASMCs) under hypoxic conditions and so could prove therapeutic targets for hypoxia-induced pulmonary hypertension [32]. SOCE and basal [Ca^2+^] are increased in high fat-fed apolipoprotein E-knockout mice, which have elevated blood lipids but no atherosclerotic plaques [33], and ORAI1 knockdown or chemical inhibition reduced atherosclerotic plaque size in this model [34] suggesting a potential therapeutic role to treat atherosclerosis and neointimal hyperplasia [35,36]. SOCE in platelets is required for thrombus formation [37] making it a target for the development of antithrombotic drugs [38,39]. Several small molecule SOCE inhibitors have been shown to reduce thrombus formation in whole blood, and 2-APB reduced thrombus formation in a murine stroke model [40]. These are thought to function by inhibition at ORAI1, although this remains to be definitively proven. Other potential therapeutic targets for SOCE inhibitors include skeletal muscle diseases [41], cancers [42–44], neurology and pain [45–48] and secretory epithelial cell disorders [49–51]. Although none are currently licensed for clinical use, a number have reached clinical trials for conditions including acute pancreatitis and COVID-19-associated pneumonia [52–54]. The success of these compounds in reaching clinical trials highlights the plausibility of SOCE inhibition as a safe therapeutic strategy in humans.

While the potencies of several SOCE inhibitors are published, they have been studied by different research groups/companies using different experimental approaches and cell lines for assessment. A direct comparison between the literature compounds using a single assay and a non-disease specific cell line is lacking. The existing SOCE inhibitors have been reviewed extensively and recently [36,55–59]. Here we report a direct comparison of the published SOCE small molecule inhibitors (presented in S1 Fig) using a fluorescence based Ca^2+^ recording system to measure thapsigargin (TG) induced SOCE in Human Embryonic Kidney 293 (HEK293) cells. These Ca^2+^ events were measured with Fura-2, a ratiometric dye that is a popular tool for the study of Ca^2+^ events due to its brightness, resistance to photobleaching, high affinity for Ca^2+^, and its selectivity over other divalent cations [60,61]. Thapsigargin, a widely used natural SERCA pump inhibitor with high potency and selectivity, was used to deplete the ER Ca^2+^ stores in the absence of extracellular Ca^2+^, before extracellular Ca^2+^ was added back, triggering SOCE [62,63]. HEK293 cells are frequently used for the initial assessment of SOCE inhibitors. Recent studies in which knock-out of ORAI1, 2 or 3 either alone or in combination have clearly demonstrated ORAI1 to be the main store-operated channel in this cell line [64,65] making it an excellent model system for screening compounds with potential for therapeutic use.

## Materials and methods

### Cell culture

HEK293 cells (CRL-1573, ATCC, Teddington, UK) were maintained in Dulbecco’s Modified Eagle’s Medium (DMEM, Gibco, Thermo Fisher Scientific, UK) supplemented with 10% fetal bovine serum (FBS, Gibco, Thermo Fisher Scientific, UK) and 100 unit mL-1 penicillin-streptomycin (Gibco, Thermo Fisher Scientific, UK) at 37°C in a humidified 5% CO_2_ incubator. Cells were grown to 95% confluence before passage and utilised at 95% confluence for experiments. Cells were used up to passage number ~30 before being discarded.

### Chemicals

All chemicals and solvents were used as supplied. SKF96365 (1), CAI (2), 2-APB (3), Pyr2 (4), Pyr3 (5), Synta-66 (6), RO2959 (7) AnCoA4 (10), leflunomide (15), Gd^3+,^ (18) and La^3+^ (19) were purchased from Sigma-Aldrich (UK). CM4620 (14) was purchased from MedChemExpress (Insight Biotechnology Ltd, UK). Teriflunomide (16) was purchased from APExBIO (UK). MRS1845 (11) was purchased from Santa Cruz Biotechnology (Insight Biotechnology Ltd, UK). Compounds that were not commercially available were synthesised. CM3457 (13) was synthesised by Dr Rajendra Gosain (unpublished). JPIII (17) was synthesised as previously reported [31]. The synthetic route for GSK7975A (9) and GSK5503A (8) largely followed the patent protocol and is summarised in S2 and S3 Figs, respectively [66]. Synthesis of the 7-azaindole series compound (12) (S4 Fig) loosely followed the patent protocol [67]. Detailed chemistry methods for compound syntheses can be found in S1 Appendix and NMR data for all compounds and intermediates synthesized can be found in S5–S17 Figs.

### Fura-2 Ca^2+^ addback assay

**Fig 1:**
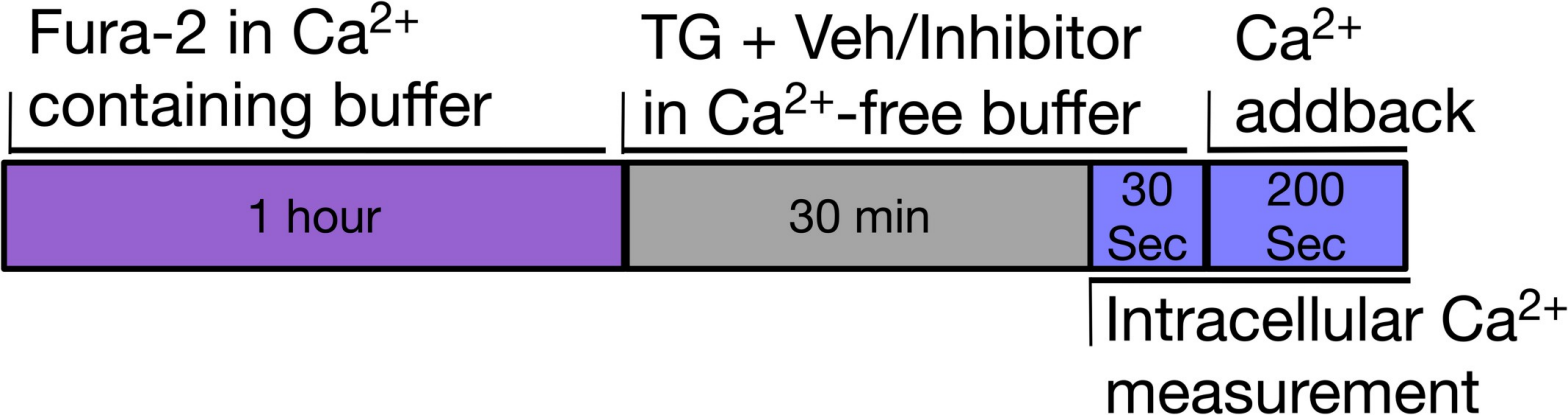
A time series schematic for the Ca^2+^ addback protocol.

Fluorescence measurements were recorded using a FlexStation III (Molecular Devices Limited, UK), running software Softmax Pro version 4.7.1 or 7.0.3. HEK293 cells were seeded onto Cellcoat Poly-D-Lysine 96-well plates (Greiner, UK) at a seeding density of 60,000 cells per well and incubated overnight at 37°C in a 5% CO_2_ incubator. The Ca^2+^ addback protocol used here followed published protocols [31,68,69]. Briefly, cells were incubated in 1.5 mM Ca^2+^ SBS (standard bath solution, NaCl 135 mM, KCl 5 mM, MgCl_2_ 1.2 mM, Glucose 8 mM, HEPES 10mM, CaCl_2_ 1.5 mM, pH 7.4) containing Fura-2AM (Molecular Probes, Thermo Fisher Scientific, UK) (2 μM) and 0.01% pluronic acid (in DMSO) for 1 hour at 37°C in the dark and then washed with 1.5 mM Ca^2+^ SBS. Thapsigargin (Sigma, UK) (1 μM) and test compound (concentrations below) or vehicle in 0 mM Ca^2+^ SBS (NaCl 135 mM, KCl 5 mM, MgCl_2_ 1.2 mM, glucose 8 mM, HEPES 10 mM, EGTA 0.4 mM, pH 7.4) were added and the cells incubated for a further 30 minutes at room temperature in the dark. Afterwards, 1.5 mM Ca^2+^ SBS, also containing test compound at the same concentration as the pre-treatment, was added to enable SOCE upon Ca^2+^ addback (0.3 mM final Ca^2+^ addback concentration after the 1:5 dilution). The 0.3 mM Ca^2+^ addback concentration was selected based on the demonstration that subtle changes in SOCE may be masked when higher Ca^2+^ addback concentrations are used [4]. Fluorescence measurements were recorded every 5 seconds for 200 seconds using 340/380 nm excitation wavelength and 510 nm emission. A schematic of this process is shown in Fig 1. AnCoA4 (10) and MRS1845 (11), were also tested with a longer exposure to compound, via inclusion of compound during the 1 hour Fura-2 loading step, such that a total exposure time of 90 mins was tested to determine whether a longer preincubation improved potency.

### Calculations and statistics

The ratio between excitation at 340 nm and 380 nm was calculated at each timepoint (ΔF^340/380^). The mean of the first three readings was used as the baseline and subtracted from each value to calculate the baseline corrected ΔF^340/380^. The IC_50_ curves were calculated using the peak baseline corrected ΔF^340/380^ at concentrations of 0.1 nM, 1 nM, 10 nM, 100 nM, 1 μM and 10 μM [compound] after subtraction of the no TG control peak value. Values were then normalized to the vehicle containing TG and a modified Hill equation was fitted to the data. The exceptions to this (where literature indicated that the standard concentration range would be inappropriate due to reduced compound potency) were: SKF96365 (1) and AnCoA4 (10) (assessed at 2.5, 5, 10, 20, 40 and 80 μM); Carboxyamidotriazole (2) (assessed at 2 nM, 20 nM, 200 nM, 2 μM, 10 μM and 20 μM); 2-APB (3) (assessed at 5, 10, 25, 33, 66, and 100 μM); MRS1845 (11) (assessed at 1 nM, 10 nM, 100 nM, 1 μM, 10 μM and 50 μM); CM4620 (14) (assessed at 0.1 nM, 1 nM, 10 nM, 100 nM, 1 μM and 5 μM); leflunomide (15) (assessed at 10, 100, 200, 300, 400 and 500 μM); teriflunomide (16) (assessed at 10, 50, 100, 200, 300 and 400 μM); JPIII (17) (assessed at 20 nM, 70 nM, 300 nM, 1.25 μM, 5 μM and 20 μM). A DMSO concentration of 0.1% v/v was consistent in all solutions. The mean ± SD was calculated from three independent experiments (n = 3), with each condition tested in triplicate for each experiment (N = 3/n = 3). To perform a comparison of SOCE inhibitory activity the % vehicle of each inhibitor at 10 μM was analyzed using an ANOVA one-way test with Dunnett’s post-hoc test using GraphPad Prism 9.0. A p value < 0.05 was considered significant.

## Results

### Evaluation of the SOCE inhibitors in HEK293 cells

**Fig 2:**
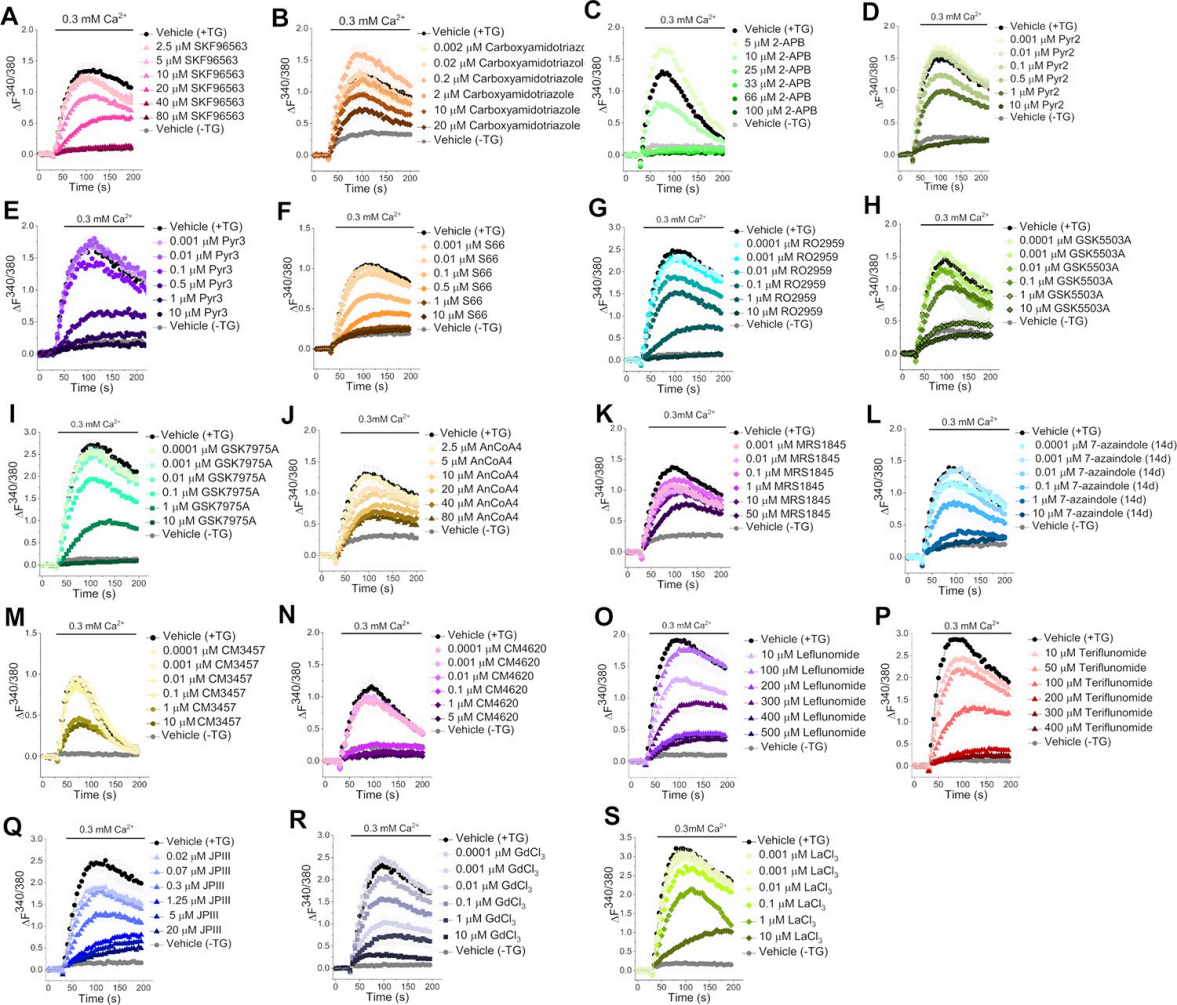
Example fluorescence versus time graphs for each of the inhibitors studied at the concentrations used to generate the dose response curves (Fig 3). ΔF^340/380^ refers to the ratio of fluorescence emission following excitation at 340 nm and 380 nm. Data has been baseline corrected to zero. Ca^2+^ addback is initiated at t = 30 seconds. Data points are presented as mean ± SEM (n = 3).

**Fig 3:**
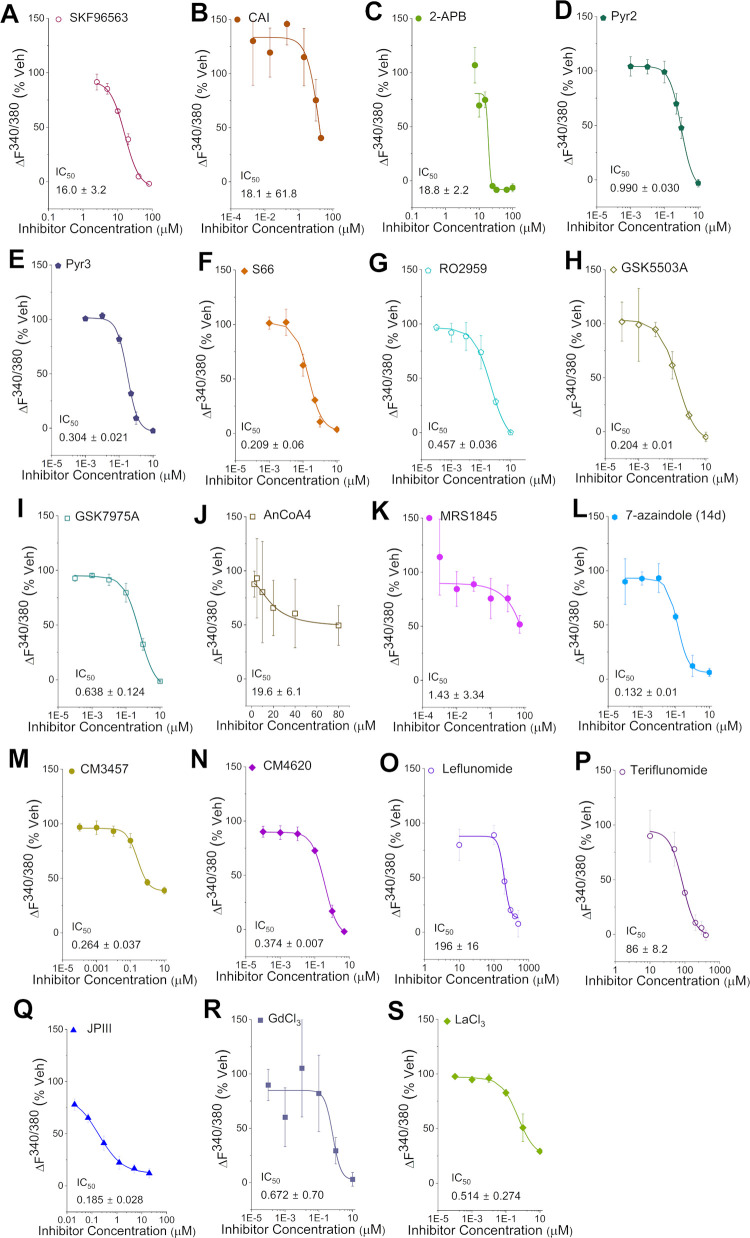
IC_50_ curves for SOCE inhibitory activity of the compounds profiled. Data presented as mean ± SD (n = 3). IC_50_ value derived from a fitted Hill1 equation.

The TG induced SOCE method was used to monitor ΔF^340/380^ fluorescence over time as a proxy for intracellular Ca^2+^ concentration (Fig 2) and used to generate dose response curves for each compound (Fig 3) with a standard pre-incubation of 30 mins. All data used for subsequent IC_50_ calculations can be found in S1 Dataset.

SKF96365 (1) was originally identified as an inhibitor of receptor-mediated Ca^2+^ entry [70], and of nonselective cation currents [71]. It is better known as a TRP channel inhibitor [72,73], and it also inhibits T-type Ca^2+^ channels [74]. It reportedly inhibits SOCE with an IC_50_ of 4 μM in patch clamp studies of rat peritoneal mast cells using Fura-2 as a Ca^2+^ indicator [75]. It has also been reported to inhibit SOCE with an IC_50_ of 12 μM in Jurkat E6-1 lymphocytes, using the Indo1 dye in a fluorescence assay, which was supported by patch clamp data [76]. Under our assay conditions SKF96365 (**1**) showed almost complete block of SOCE at 40 μM, but only partial block at 10 μM (Fig 2A), which generated an IC_50_ of 16 μM (Fig 3A), roughly consistent with the previously published values observed in Jurkat T cell assays.

Carboxyamidotriazole (CAI, 2) was originally identified as a Ca^2+^ influx inhibitor. It is also a general inhibitor of non-voltage gated Ca^2+^ influx [77]. A dose-dependent reduction in Ca^2+^ influx by CAI has been reported using an assay setup similar to ours, with an IC_50_ of 0.5 μM when a 5-minute pre-incubation was used [78]. In our hands, CAI (2, used as the free amine) showed partial inhibition of SOCE in HEK293 cells at 10 μM with better inhibition at 20 μM, the highest dose tested (Fig 2B). We therefore generated an IC_50_ of 18.1 μM (Fig 3B), which was significantly higher than the published values.

2-APB (3) was originally identified as an inhibitor of inositol trisphosphate (IP_3_)-modulated Ca^2+^ release [79], but it has a less straightforward effect on SOCE than other inhibitors–the current is rapidly enhanced at low concentrations (~1–5 μM), but is inhibited at ≥10 μM in Jurkat T cells, RBL cells and DT40 B lymphocytes [80]. 2-APB was challenging to work with due to its activating effect at the lowest concentrations we tested (Fig 2C). It was difficult to record complete inactivity and therefore generating a reliable IC_50_ curve for 2-APB (3) was challenging (Fig 3C). In our hands the IC_50_ of 2-APB was calculated as ~19 μM which was three to five-fold less potent than published for CHO, HeLa and DT40 cells [81].

The 3,5-bis(trifluoromethyl)pyrazole (BTP) series were identified from a high-throughput screen to find novel IL-2 inhibitors for clinical use as immunosuppressants [82]. They are believed to block the nuclear import of nuclear factor of activated T-cells (NFAT) and thereby prevent NFAT-dependent transcription of pro-inflammatory genes. BTP2, (also known as Pyr2) was identified from this study [83]. Pyr2 was found to inhibit SOCE in Jurkat T cells with an IC_50_ of 10–100 nM, depending on the incubation time [84,85]. A different publication reported IC_50_ values of 0.59 μM for Pyr2 (4) and 0.54 μM for its sister compound, Pyr3 (5), for endogenous SOCE in RBL-2H3 cells [86]. In our hands, Pyr2 (4) showed complete inhibition of SOCE at 10 μM (Fig 2D), resulting in an IC_50_ of 990 nM (Fig 3D). Its close relative Pyr3 (5) showed very similar behaviour with slightly more potent block of SOCE at 1 μM concentration (Fig 2E), producing an IC_50_ of 304 nM (Fig 3E). Our Pyr2 (4) IC_50_ appears a little higher than the previously reported values, whilst our Pyr3 (5) IC_50_ is in agreement with published IC_50_ values recorded in RBL-2H3 cells [86].

Synta Pharmaceuticals (now Madrigal Pharmaceuticals) hold a patent on a series of SOCE inhibitors for immune and inflammatory disorders [87]. Lead compound Synta-66 (6) has been screened against a commercial panel of membrane proteins and ion channels and no significant activity was found [24]. It is a moderately potent SOCE inhibitor in blood cells, with IC_50_ values of ~1 μM in Jurkat T [24], 1.4 μM in RBL [24] and 3 μM in RBL-1 cells [88], respectively. However, it is highly potent in VSMCs and HUVECs, with an IC_50_ of ~26 nM for both [68,69] and shown to have no effect on STIM1/STIM1 clustering, TRP channels or the nonselective cationic current in VSMCs, which implies that it is not a general ion channel inhibitor [68,69] Synta-66 (6) was moderately potent showing almost complete block at 1 μM (Fig 2F). The IC_50_ of 209 nM (Fig 3F) falls well within the range of IC_50_ values obtained across a range of cell types.

Hoffmann-La Roche holds patents on SOCE inhibitors for the treatment of immune or inflammatory disorders, one of which claims their lead compound RO2959 (7) to have an IC_50_ of 15 nM for IL-2 inhibition [89]. It had an IC_50_ for SOCE inhibition of 402 nM in RBL-2H3 and 265 nM in CD4^+^ T cells [90]. RO2959 demonstrated an IC_50_ of 25 nM for T-Rex-CHO (Chinese hamster ovary) cells overexpressing ORAI1/STIM1 vs. 530 nM in ORAI3/STIM1 overexpressing cells as measured by patch clamp [90], which represents a 20-fold selectivity ratio for ORAI1. RO2959 (7) in our hands produced complete inhibition at 10 μM (Fig 2G) producing an IC_50_ of 457 nM (Fig 3G) which are consistent with data obtained from RBL-2H3 cells and CD4+ T-cells [90].

GlaxoSmithKline (GSK) holds a patent on a series of pyrazole-based SOCE inhibitors for the treatment of allergic and immune disorders [66]. GSK5503A (8) and GSK7975A (9) were confirmed to block Ca^2+^ currents at 10 μM in patch clamp experiments in HEK293 cells [91]. GSK7975A was further characterized to demonstrate a very similar IC_50_ for ORAI1 and ORAI3 inhibition (4.1 vs 3.8 μM), but GSK5503A was not further studied [91]. This study also found them to have no effect on STIM1/STIM1 clustering or the STIM1/ORAI1 interaction, implying an extracellular binding site. This finding is supported by the reduced ability of GSK7975A to inhibit SOCE against the ORAI1 E106D pore mutant (reduced Ca^2+^ selectivity), implying that a conformational change in the glutamate selectivity filter may affect compound binding [91]. GSK7975A was screened against a small panel of ion channels and receptors overexpressed in HEK293 cells and was found to be selective against all of them (IC_50_ > 10 μM), except for an IC_50_ of 8 μM against Ca_V_1.2 [91]. In our hands, GSK5503A (8) was found to completely inhibit SOCE at 10 μM (Fig 2H) and generated an IC_50_ of 200 nM (Fig 3H). This appeared to be slightly more potent than the related compound GSK7975A (9) which also completely blocked SOCE at 10 μM (Fig 2I) with an IC_50_ of 638 nM (Fig 3I) which was in close agreement to previously reported findings of 0.8 μM in RBL-2H3 [91] and 0.5 μM in Jurkat cells, respectively [92].

AnCoA4 (10) was identified as an ORAI1 inhibitor using a commercial small molecule microarray, which screened against minimal functional domains–purified isolated domains of ORAI1 and STIM1 that are known to be vital for SOCE activity [93]. Using this technique, only small molecules that bind to ORAI1 or STIM1 should be identified as hits, avoiding indirect SOCE inhibitors. AnCoA4 has an IC_50_ of 880 nM in HEK293T cells calculated from an NFAT reporter gene luciferase assay [93]. AnCoA4 (10) failed to inhibit SOCE at 10 μM in our assay and only demonstrated partial inhibition when tested up to 80 μM (Fig 2J) producing an IC_50_ of 19.6 μM (Fig 3J). We tested AnCoA4 over the same concentration range with a slightly longer exposure time, by including the compound during the Fura-2 loading step, which yielded a marginal improvement to produce an IC_50_ of 12.5 μM (S18 Fig).

N-propargylnitrendipine (MRS1845) was identified in a screen of 1,4-dihydropyridines as potential SOCE inhibitors, demonstrating an IC_50_ of 1.7 μM in HL-60 cells although it was also shown to have a similar potency for inhibition of L-type calcium channels (IC_50_ = 2.1 μM) [94]. In our hands, MRS1845 gave approximately a 50% block at 50 μM, the highest dose tested (Fig 2K) with an IC_50_ of 1.43 μM (Fig 3K), although this should be interpreted with caution given the lack of a sigmoidal curve upon fitting the Hill1 equation. We noted a solubility issue at 50 μM in the aqueous solution and therefore retested the compound at a slightly lower concentration of 30μM, with a longer exposure time, by including compound during the Fura-2 loading step. This yielded a higher IC_50_ of 9.06 μM (S19 Fig) although the fit of the Hill1 equation is more accurate indicating that MRS1845 performs better with a longer exposure time.

Based on the structures of Pyr2 and Synta-66, a series of 7-azaindole SOCE inhibitors were developed as potential treatments for asthma [95]. Lead compound 7-azaindole 14d (12) is a potent SOCE inhibitor with an IC_50_ of 150 nM in Jurkat T cells, showing dose-dependent inhibition of eosinophils in a rat model of allergic respiratory inflammation [95]. In our hands 7-azaindole 14d (12) showed strong, but not complete inhibition at 10 μM, (Fig 2L). The IC_50_ was calculated as 132 nM (Fig 3L) which was almost in complete agreement with the IC_50_ generated with Jurkat cells in a similar assay set-up [95].

CalciMedica holds several patents on CRAC inhibitors. Their lead compound CM4620 (14) [96], has reportedly completed Phase II clinical trials for treatment of acute pancreatitis with systemic inflammatory response syndrome (SIRS), and others are ongoing [53,97–99]. A related compound, CM3457 (13), has shown immunomodulatory effects in different cell lines, including interleukin inhibition [100] and was shown to be selective for ORAI1 inhibition over several other K^+^, Na^+^ and Ca^2+^ channels [100]. CM4620 (14) has an IC_50_ of ~0.1 μM in ORAI1/STIM1 overexpressing HEK293 cells, measured by whole-cell patch clamp experiments, as well as ~0.7 μM in murine pancreatic acinar cells [101]. The related compound, CM3457 (13), potently inhibits endogenous SOCE in RBL-2H3 cells with an IC_50_ of 25 nM and Jurkat T-cells with an IC_50_ of 17 nM, which were comparable to the IC_50_ of 34nM observed for HEK293 cells stably overexpressing ORAI1/STIM1 [100]. Of the CalciMedica inhibitors, CM4620 (14) showed almost complete inhibition of SOCE at 10 μM (Fig 2N) generating an IC_50_ of 374 nM (Fig 3N). This is in good agreement with a previously observed IC_50_ of ~0.1 μM in ORAI1/STIM1 overexpressing HEK293 cells [101]. The older compound CM3457 (13) failed to show complete SOCE inhibition at 10 μM (Fig 2M) and therefore the calculated IC_50_ of 264 nM may be an overestimate of its potency (Fig 3M).

Several FDA-approved drugs have been identified as weak to moderately potent SOCE/ORAI1 inhibitors in a single study, using a ligand-based virtual screen [102]. Five drugs (leflunomide, teriflunomide, tolvaptan, lansoprazole and roflumilast) showed substantial (≥60%) SOCE inhibition at 10 μM, and a dose-response curve was generated. Leflunomide (15) and its active metabolite teriflunomide (16) showed SOCE inhibition at clinically relevant concentrations, with IC_50_ values of ~10 μM (leflunomide) and ~21 μM (teriflunomide), respectively [102], which is lower than most other reported inhibitors. Leflunomide is approved as a dihydro-orotate dehydrogenase inhibitor used to treat rheumatoid arthritis and psoriatic arthritis [103]; teriflunomide was later approved for the treatment of multiple sclerosis [104]. Under our conditions leflunomide (15) and teriflunomide (16) showed no inhibition at 10 μM, so the compounds were tested up to 500 μM and 400 μM, respectively. Partial SOCE inhibition was observed with 400 and 500 μM leflunomide (15) (Fig 2O) generating an IC_50_ of 196 μM (Fig 3O). Teriflunomide (16) was more potent providing more of a dose-response with strong inhibition above 200 μM (Fig 2P) generating an IC_50_ of 86 μM (Fig 3P), a value significantly less potent than previously quoted IC_50_ values.

Based on the structure of Synta-66, JPIII (17) was recently developed in-house as a more water-soluble alternative [31]. It is moderately potent and highly selective for ORAI1 over other Ca^2+^-permeable channels and was well-tolerated and effective in a mouse model of cardiac hypertrophy [31] and rat model of pulmonary hypertension [105]. Overexpression of ORAI1 did not affect the potency of JPIII, only the amplitude of the fluorescence signal due to increased [Ca^2+^] [31]. JPIII (17) demonstrated partial SOCE inhibition in a dose-response manner (Fig 2Q) with an IC_50_ of 185 nM (Fig 3Q), similar to the IC_50_ of 399 nM previously reported in HEK293 cells [31].

The trivalent lanthanide cations, such as gadolinium (Gd^3+^) (18) and lanthanum (La^3+^) (19) are known to block SOCE in the low micromolar range, with 1 μM Gd^3+^ causing complete channel block in a range of cell lines [106–108]. Gd^3+^ inhibits SOCE with an IC_50_ of 34 nM in the rat smooth muscle A7r5 cell line [107]. La^3+^ has been found to potently inhibit SOCE in Jurkat T cells stimulated with either thapsigargin or CD3 monoclonal antibody with an IC_50_ of 20 nM [106]. Gd^3+^ (18) showed moderate inhibition of SOCE at 10 μM (Fig 2R), whilst La^3+^ (19) demonstrated partial inhibition of SOCE at 10 μM (Fig 2S). These generated IC_50_ values of 0.67 μM (Fig 3R) and ~0.5 μM (Fig 3S), respectively, which were around 20-fold less potent than reported in the literature.

**Fig 4:**
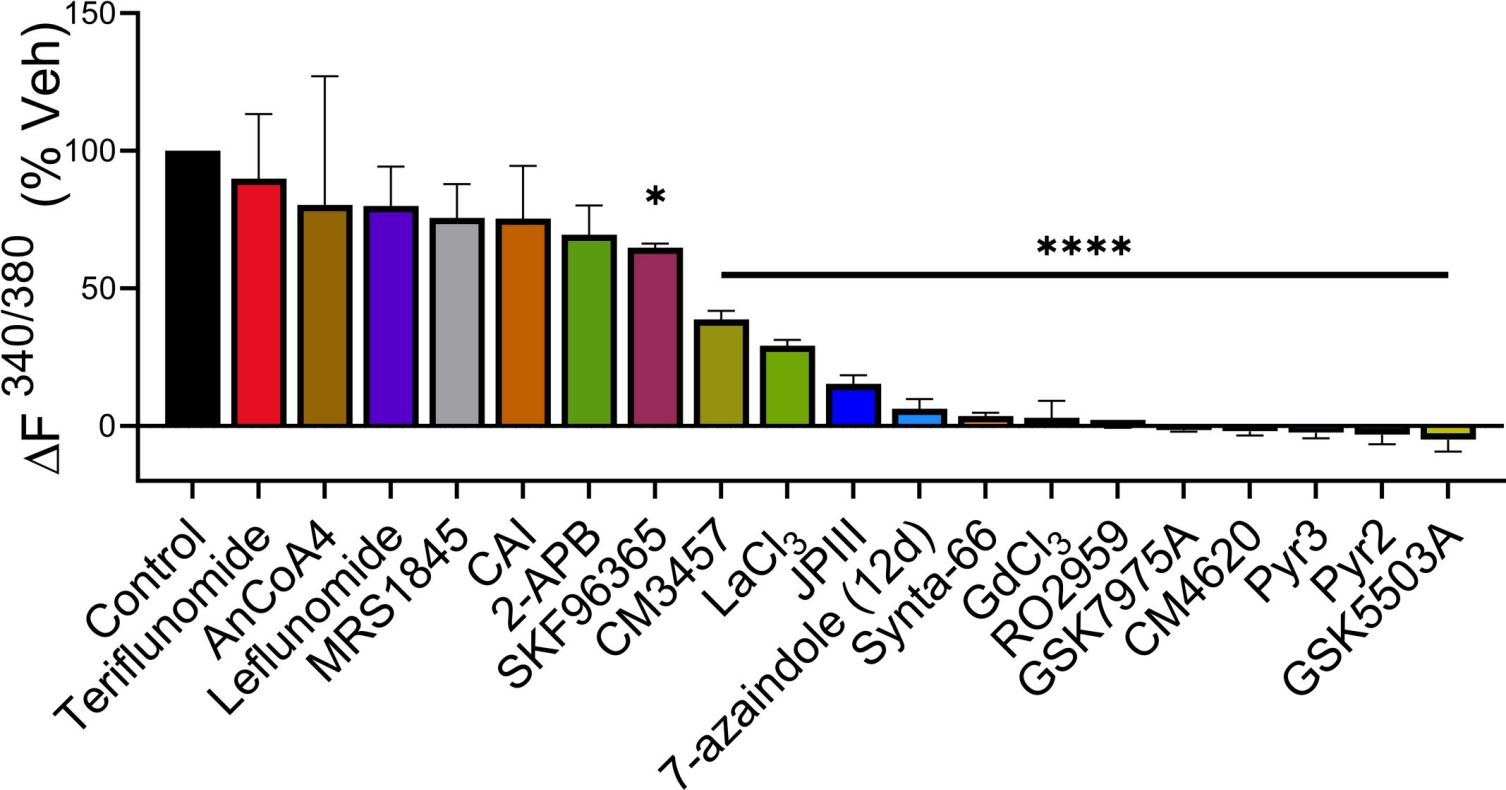
A comparison of the SOCE inhibitory activity as % vehicle of each inhibitor at 10 μM in the Ca^2+^ addback assay. Statistical significance was calculated using an ANOVA one-way test with Dunnett’s post-hoc test, where *p<0.05, and ****p<0.0001 (n = 3).

To allow a cross-wise comparison of the potency of the compounds we calculated the SOCE inhibitory activity as % vehicle of each inhibitor at 10 μM in Fig 4. With the exception of CAI (2), 2-APB (3), AnCoA4 (10), MRS1845 (11), leflunomide (15) and teriflunomide (16) all tested compounds showed significant SOCE inhibitory activity at this concentration. For ease of reference our calculated IC_50_ values are shown alongside comparable values obtained from the literature in Table 1.

**Table 1:** SOCE inhibitory activity (IC_50_ ± SE) of the SOCE inhibitors studied, compared to their reported IC_50_ values.

Compound name	Residual Ca^2+^ entry at 10μM	IC_50_ (HEK293) / μM ± SE	Reported SOCE inhibition IC_50_ / μM	Cell line for reported IC_50_
SKF96365 (**1**)	64.9%	16.0 ± 1.8	4 12	rat peritoneal mast cells [75] Jurkat E6-1 lymphocytes [76]
CAI (**2**)	75.4%	18.1 ± 61.8	0.5	HEK293 [78]
2-APB (**3**)	69.5%	18.8 ± 2.2	2.9 ± 0.1 4.8 ± 0.6 6.5 ± 0.3	CHO [81] IP_3_R-knockout DT40 [81] HeLa [81]
Pyr2 (**4**)	0%	0.990 ± 0.030	0.01–0.1 0.59	Jurkat T cells [84, 85] RBL-2H3 [86]
Pyr3 (**5**)	0%	0.304 ± 0.021	0.54	RBL-2H3 [86]
Synta-66 (**6**)	3.6%	0.209 ± 0.06	0.026 0.026 1.4 ~1 3	VSMC [68,69] HUVEC [68,69] RBL [24] Jurkat T cells [24] RBL-1 [88]
RO2959 (**7**)	0%	0.457 ± 0.036	0.265 ± 0.016 0.402 ± 0.129	human CD4^+^ T-cells [90] RBL-2H3 [90]
GSK5503A (**8**)	0%	0.204 ± 0.01	Full block at 10 μM	HEK-293 overexpressing ORAI1/STIM1 [91]
GSK7975A (**9**)	0%	0.638 ± 0.124	0.5 0.8 ± 0.1	human CD4^+^ T-cells [92] RBL-2H3 [91]
AnCoA4 (**10**)	95.8%	19.6 ± 6.1	0.88	IC_50_ in HEK293T, NFAT inhibition [93]
MRS1845 (**11**)	75.7%	1.43 ±??	1.7	HL60 cells [94]
7-azaindole (**12**)	6.2%	0.132 ± 0.01	0.150 ± 0.022	Jurkat T cells [95]
CM3457 (**13**)	38.8%	0.264 ± 0.037	0.017 0.025 0.034	Jurkat T cells [100] RBL-2H3 [100] HEK293 overexpressing ORAI1/STIM1 [100]
CM4620 (**14**)	0%	0.374 ± 0.007	0.1 0.7	HEK293 overexpressing ORAI1/STIM1 [101] human pancreatic acinar cells [101]
Leflunomide (**15**)	80%	196 ± 16	~10	RBL-1 [102]
Teriflunomide (**16**)	90%	86 ± 8.2	~21	RBL-1 [102]
JPIII (**17**)	15.3%	0.185 ± 0.028	0.399	HEK293 [31]
Gd^3+^ (**18**)	2.96%	0.672 ± 0.70	0.034 ± 0.005	A7r5 [107]
La^3+^ (**19**)	28.5%	0.514 ± 0.274	0.02	Jurkat T cells [106]

Reported inhibition refers to endogenous SOCE unless stated. Literature inhibition values only include those from similar cell-based Ca^2+^ addback assays, with standard error reported where given. All IC_50_ values were generated in native HEK293 cells (n = 3).

## Discussion

A variety of reported SOCE inhibitors were tested in a standard HEK293 cell Ca^2+^ addback assay with Fura-2 as the indicator dye and thapsigargin to deplete stores. Native HEK293 cells were chosen as a model system to study SOCE, as the Ca^2+^ addback response following TG depletion has been demonstrated to be highly specific to ORAI1 [64]. Knockout of ORAI1, either alone or in combination with ORAI2 or 3 knockout completely ablated Ca^2+^ entry whilst knockout of either ORAI2, ORAI3 or double knockout of ORAI2/3 had no effect on Ca^2+^ entry in a similar assay [64] Interestingly, overexpression of ORAI2 in ORAI1/2/3 triple knockout cells lead to a greater influx of Ca^2+^ compared to native cells, which the authors reasoned was due to the much lower expression of ORAI2 and ORAI3 compared to ORAI1 in native HEK293 cells [64]. Differing IC_50_ values have been reported for compounds between native HEK293 cells and those overexpressing ORAI1 and/or STIM1 [93]. These studies highlight the caution needed when extrapolating data from overexpression systems to the native *in vivo* environment. Under our assay conditions most compounds showed significant SOCE inhibition. We were able to demonstrate that the most potent inhibitors in this side-by-side comparison were: 7-azaindole 14d (12), JPIII (17), Synta-66 (6), Pyr 3 (5), GSK5503A (8), CM4620 (14) and RO2959 (7)—all giving IC_50_ values < 0.5μM. Of the compounds tested: SKF96365 (1), Pyr3 (5), Synta-66 (6), RO2959 (7), GSK7975A (9), MRS1845 (11), 7-azaindole 14d (12), CM4620 (14), and JPIII (17) generated IC_50_ values consistent with the literature. Conversely, CAI (2), 2-APB (3), Pyr2 (4), AnCoA4 (10), CM3457 (13), Leflunomide (15), Teriflunomide (16), Gd^3+^ (18) and La^3+^ (19) generated IC_50_ values greater than published values.

In our assay, CAI (2) did not demonstrate full inhibition of SOCE at 20 μM, the highest dose tested. This was surprising given that a one hour pretreatment with 5 μM CAI inhibited SOCE in three different ovarian cancer cell lines using a similar assay setup [109] and in another study an IC_50_ of 0.5 μM was generated when a 5-minute pre-incubation was used [78]. The authors noted inhibition to be time-dependent, with 40% inhibition seen at 10 μM after a 10-second pre-incubation with CAI, compared with complete block after 5 minutes [78]. The time-dependence was also confirmed by whole-cell patch clamp experiments, and it was suggested to act via a complex mechanism affecting mitochondrial membrane polarisation rather than as a simple ion channel blocker [78]. In our assay setup cells were pre-incubated with test compound for 30 minutes, which should be sufficient to observe an effect for CAI based on the literature.

2-APB (3), proved technically challenging to work with due to its well established activating effect at the lowest concentrations tested and inhibitory effect at higher concentrations [80]. Given these difficulties the IC_50_ we recorded was -three to -fivefold less potent than published values. The effects of 2-APB are variable between ORAI1/ORAI2/ORAI3 [110]. 2-APB can interact with other ion channels and receptors that influence SOCE, including TRP channels, the IP_3_ receptor and the SERCA pump [111], alongside ORAI2 and ORAI3 [112].

Pyr2 (4) was initially described as the first selective CRAC channel inhibitor [83,85] but was later found to inhibit TRPC3 and TRPC5 channels, and to activate TRPM4 [113,114]. It has been proposed to inhibit from the extracellular space based on patch clamp experiments [85]. Our IC_50_ value of ~1 μM was higher than the range of values reported (10–590 nM) [84–86,90], possibly due to the difference in incubation time. In one study the cells were incubated in test compound for a few minutes [84], whereas in another study they were incubated for 24 hours, with maximal block seen after 2 hours [85].

AnCoA4 (10) had a reported IC_50_ of 880 nM (HEK293T), as measured using an NFAT reporter gene luciferase assay, which is an indirect measure of SOCE using a downstream signalling pathway [93]. It has been validated as a SOCE inhibitor by other groups [115–117], and also showed 80% inhibition of Ca^2+^ current (20 μM) in patch clamp studies of ORAI1 [93], but a dose-response for direct inhibition of SOCE was not determined in any of these studies. AnCoA4 was proposed to act intracellularly by blocking the ORAI1/STIM1 interaction [93], Repurchased AnCoA4 failed to completely inhibit SOCE in our assay at 80 μM, the highest dose tested. In the NFAT luciferase assay, the cells were pre-incubated with AnCoA4 for 6 hours before initiation of SOCE, whereas our assay only has a 30-minute compound incubation step. We tested AnCoA4 with a longer pre-incubation by adding AnCoA4 during the Fura-2 loading step such that cells were exposed to the compound for 90 mins in total. We observed a modest improvement in IC_50_ with the longer pre-incubation, but still did not achieve complete SOCE inhibition at the highest dose.

MRS1845 (11) was previously shown to have an IC_50_ of 1.7 μM in HL-60 cells [94], but ATP was used as the stimulus for SOCE instead of thapsigargin. In our hands, MRS1845 achieved approximately a 50% block of SOCE at 50μM with little to no block at 10 μM, although we noted solubility issues at the higher concentrations in aqueous solution. Our results are in agreement with a lack of potency observed against convulxin/thrombin stimulated SOCE in platelets at concentrations up to 100 μM [118]. Others have reported potency of MRS1845 against thapsigargin-induced SOCE in a Fura-2 based assay in both ovarian carcinoma cells [119] and human aortic smooth muscle cells [120,121] although exposure to MRS1845 was for 24 hours indicating it may act on SOCE in an indirect manner.

In our hands, CM3457 (13) failed to completely inhibit SOCE at the highest concentration tested (10μM) and was around 10-fold less potent in native HEK293 cells than observed in a Fluo-4 based assay of ORAI1 overexpressing HEK293 cells or immune cells [100]. Due to the low amplitude of the I_CRAC_ current, overexpressing cells are often used to increase the amplitude of the addback response by fluorescence imaging for ease of measurement and this could account for the observed difference. The FDA-approved drugs although still active in our assay were significantly less potent–leflunomide (16) by ~10-fold and teriflunomide (17) by ~4-fold. This is a large discrepancy when the only major difference is the cell line (RBL-1), -assay, pre-incubation times, dye and the mode of store depletion were similar [122].

The trivalent lanthanide cation Gd^3+^ (18) inhibits SOCE with an IC_50_ of 34 nM in the rat smooth muscle A7r5 cell line [107]. This study used a similar assay setup, with thapsigargin and Fura-2, although the imaging technique and cell line differed [107]. It also inhibited with a similar potency (IC_50_ = 50 nM) in patch clamp studies in the Drosophila S2 cell line [123]. La^3+^ (19) has been found to potently inhibit SOCE in Jurkat T cells stimulated with either thapsigargin or CD3 monoclonal antibody with an IC_50_ of 20 nM [106]. This assay used a fluorescence-activated cell sorter (FACS)-based measurement system with Indo-1 as the indicator dye, which could account for the observed difference in potency. Both Gd^3+^ and La^3+^ are less potent than the literature value in our hands, by around 20-fold, but both still retain their characteristic sub-micromolar potency. Lanthanides are not cell-permeable, and so are believed to act by simply blocking the pore [106]. However, they may act at a different binding site to Ca^2+^, as no change in La^3+^ block is seen for the well-established E106D pore mutant, which is characterised by a loss of Ca^2+^ selectivity [124]. Whilst lanthanides are widely used to study channel blockade *in vitro*, they would not be useful therapeutically as they block a variety of cation channels, and various toxic effects have been observed [125].

Although approximately half of the compounds tested were confirmed to display similar potencies to those recorded in the literature there were discrepancies in potency observed for the remaining compounds. This could be due to differences in assay setup, choice of fluorescent dye, equipment used to quantify [Ca^2+^], or differences in SOCE pathways in different cell lines. As compound binding to ORAI1 has not been empirically proven for most inhibitors, it is possible that some act upstream of the SOCE pathway rather than directly targeting it. This could also be the reason some compounds require longer pre-incubation periods and so did not appear to be active in this assay. This highlights the difficulty in developing a ‘one-size-fits-all’ assay, as a short pre-incubation step may lead to compounds with a slower mechanism of action being missed during screenings, but longer pre-incubations may not be feasible for a cell-based assay as extended Ca^2+^ depletion is cytotoxic.

The choice of cell line is likely to be an important reason for variability in potency data. Studies using RBL cells for SOCE assays are often inconsistent with the results acquired here in HEK293 cells. Synta-66 (6) is much less potent in RBL-1 cells than in HEK293 cells (3 μM vs. 130 nM). In the related line RBL-2H3, Pyr3 (5), RO2959 (7) and GSK7975A (9) show comparable potency to that observed in HEK293 cells, although CM3457 (13) is around 10-fold less potent (0.264 vs. 0.025 nM). Native RBL-2H3 cells express higher levels of ORAI1 and ORAI2 than HEK293 and they thus demonstrate a larger SOCE amplitude [126]. There may also be differences between species–HEK293, Jurkat T, HeLa and CD4^+^ cells are human cell lines, while RBL cells are from rats, CHO cells from hamsters and DT40 cells from chickens. GSK7975A (9) and its analogue GSK5498A have been previously observed to have species-dependent effects. They inhibit production of mast cell mediators and pro-inflammatory T cell cytokines in human and rat mast cells, but not in mouse or guinea pig [92]. Differences in potency of compounds may be observed between native and ORAI1-overexpressing cells, the latter being of particular use in patch clamp experiments, where the native ORAI1 current can be difficult to isolate, an issue that is not apparent when recordings are obtained from a confluent layer of cells, as occurs when using the FlexStation. Patch clamp is widely used to study ion channel inhibitor binding and was used to validate many of the profiled compounds in the literature. However, patch clamp is technically challenging and so time-consuming it is unsuitable for routine screening on drug discovery projects, although high-throughput automated patch clamp technologies have become available [127]. Although no assay is perfect the Fura-2 based assay of thapsigargin induced store depletion is well established as a reliable method of monitoring SOCE and when performed on the FlexStation III it can be used as a high-throughput screening tool. Limitations of the current study include that the TG-induced store depletion phase fell outside of the recording window as to record it would have necessitated significantly lengthening the recording window, with concomitant lengthy exposure to Ca^2+^-free conditions having an adverse effect on cell viability. Additionally, we have made the assumption that the SOCE signal was derived from activity of ORAI1 given recent reports that confirm knock-out of ORAI1 in HEK293 largely abolishes the TG-induced SOCE signal [64,65]. However, it is beyond the scope of this study to characterize all of the Ca^2+^ selective channels that could have been affected by each of the compounds.

Nonetheless, inhibitors of SOCE represent a valuable class of compounds as potential therapeutics in a variety of disease areas, including immunoinflammatory disease, cardiovascular disease and cancer. This study highlights the importance of assay design and use of biorthogonal assays to validate a novel compound. These data also provide a fair comparison of most SOCE inhibitors in a standard and well-validated assay, and so may aid others in choosing the most suitable molecule for a specific application.

## Supporting information

S1 Fig Chemical structures of the small molecule SOCE inhibitors examined in this study and their common names. (TIF)

S2 Fig Convergent synthetic route to GSK7975A (9). (TIF)

S3 Fig Synthesis of GSK5503A (8) from intermediate 21. (TIF)

S4 Fig Synthetic route to 7-azaindole (12). (TIF)

S5 Fig ^1^H NMR (400 MHz, DMSO-D_6_) (top) and ^13^C NMR (100 MHz, DMSO-D_6_) (bottom) spectra of 2,6-difluoro-*N*-(1*H*-pyrazol-3-yl)benzamide (21). (TIF)

S6 Fig ^1^H NMR (500 MHz, methanol-D_4_) (top) and ^13^C NMR (100 MHz, methanol-D_4_) (bottom) spectra of 3-trifluoromethyl-4-(hydroxymethyl)phenol (18). (TIF)

S7 Fig ^1^H NMR (400 MHz, CDCl_3_) (top) and ^13^C NMR (100 MHz, CDCl_3_) (bottom) spectra of [4-(benzyloxy)-2-(trifluoromethyl)phenyl]methanol (19). (TIF)

S8 Fig ^1^H NMR (500 MHz, CDCl_3_) (top) and ^13^C NMR (100 MHz, CDCl_3_) (bottom) spectra of N-(1-{[4-(benzyloxy)-2-(trifluoromethyl)phenyl]methyl)-1H-pyrazol-3-yl)-2,6-difluorobenzamide (22). (TIF)

S9 Fig ^1^H NMR (500 MHz, methanol-D_4_) (top) and ^13^C NMR (100 MHz, methanol-D_4_) (bottom) spectra of 2,6-difluoro-N-(1-{[4-hydroxy-2-(trifluoromethyl)phenyl]methyl}-1H-pyrazol-3-yl)benzamide (9). (TIF)

S10 Fig ^1^H NMR (500 MHz, methanol-D_4_) (top) and ^13^C NMR (125 MHz, methanol-D_4_) (bottom) spectra of 2,6‐difluoro‐*N*‐{1‐[(2‐phenoxyphenyl)methyl]‐1*H*‐pyrazol‐3‐yl}benzamide (8). (TIF)

S11 Fig ^1^H NMR (400 MHz, CDCl_3_) (top) and ^13^C NMR (100 MHz, CDCl_3_) (bottom) spectra of [(2-chloro-6-fluorophenyl)ethynyl](trimethyl)silane (25). (TIF)

S12 Fig ^1^H NMR (400 MHz, CDCl_3_) (top) and ^13^C NMR (100 MHz, CDCl_3_) (bottom) spectra of 1-chloro-2-ethynyl-3-fluorobenzene (26). (TIF)

S13 Fig ^1^H NMR (400 MHz, DMSO-D_6_) (top) and ^13^C NMR (100 MHz, DMSO-D_6_) (bottom) spectra of 5-bromo-3-[(2-chloro-6-fluorophenyl)ethynyl]pyridin-2-amine (27). (TIF)

S14 Fig ^1^H NMR (400 MHz, CDCl_3_) (top) and ^13^C NMR (100 MHz, CDCl_3_) (bottom) spectra of 5-bromo-2-(2-chloro-6-fluorophenyl)-1H-pyrrolo[2,3-b]pyridine (28). (TIF)

S15 Fig ^1^H NMR (400 MHz, CDCl_3_) (top) and ^13^C NMR (100 MHz, CDCl_3_) (bottom) spectra of 5-bromo-2,4-dimethoxypyridine (29). (TIF)

S16 Fig ^1^H NMR (400 MHz, CDCl_3_) (top) and ^13^C NMR (100 MHz, CDCl_3_) (bottom) spectra of 4,6-dimethoxypyridin-3-yl)boronic acid (30). (TIF)

S17 Fig ^1^H NMR (400 MHz, CDCl_3_) (top) and ^13^C NMR (100 MHz, CDCl_3_) (bottom) spectra of 2-(2-chloro-6-fluorophenyl)-5-(4,6-dimethoxypyridin-3-yl)-1H-pyrrolo[2,3-b]pyridine (12). (TIF)

S18 Fig Example fluorescence over time graph (A) and IC_50_ (B) for AnCoA4 following 30 minute preincubation and example fluorescence over time graph (C) and IC_50_ (D) for AnCoA4 following 90 minute preincubation.
(TIF)

S19 Fig Example fluorescence over time graph (A) and IC_50_ (B) for MRS1845 following 30 minute preincubation and example fluorescence over time graph (C) and IC_50_ (D) for MRS1845 following 90 minute preincubation.
(TIF)

S1 Appendix Detailed methods for compound synthesis. (PDF)

S1 Dataset Raw data used to calculate IC_50_ values. Baseline corrected Δ^340/380^ ratios from three independent biological replicates (Plate A, B and C) which each included three technical replicates for each condition tested.
(XLSX)
