# Supplementary figures and images for: Side-by-side comparison of published small molecule inhibitors against thapsigargin-induced store-operated Ca2+ entry in HEK293 cells
Source: PLoS One. 2024 Jan 23;19(1):e0296065. doi: 10.1371/journal.pone.0296065 (PMC10805320; doi:10.1371/journal.pone.0296065)

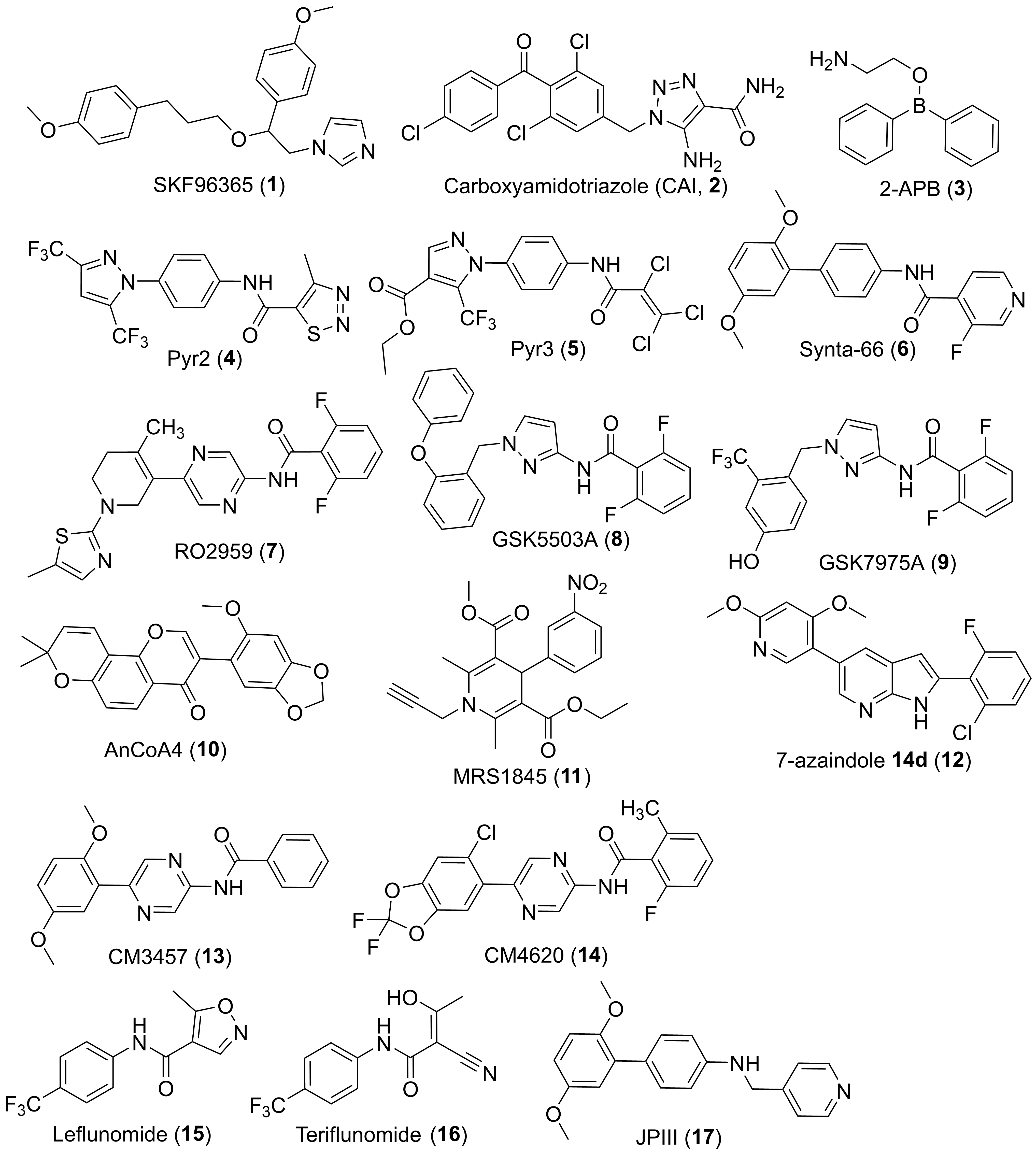

Supplement: S1 Fig — (TIF) [file pone.0296065.s001.tif]

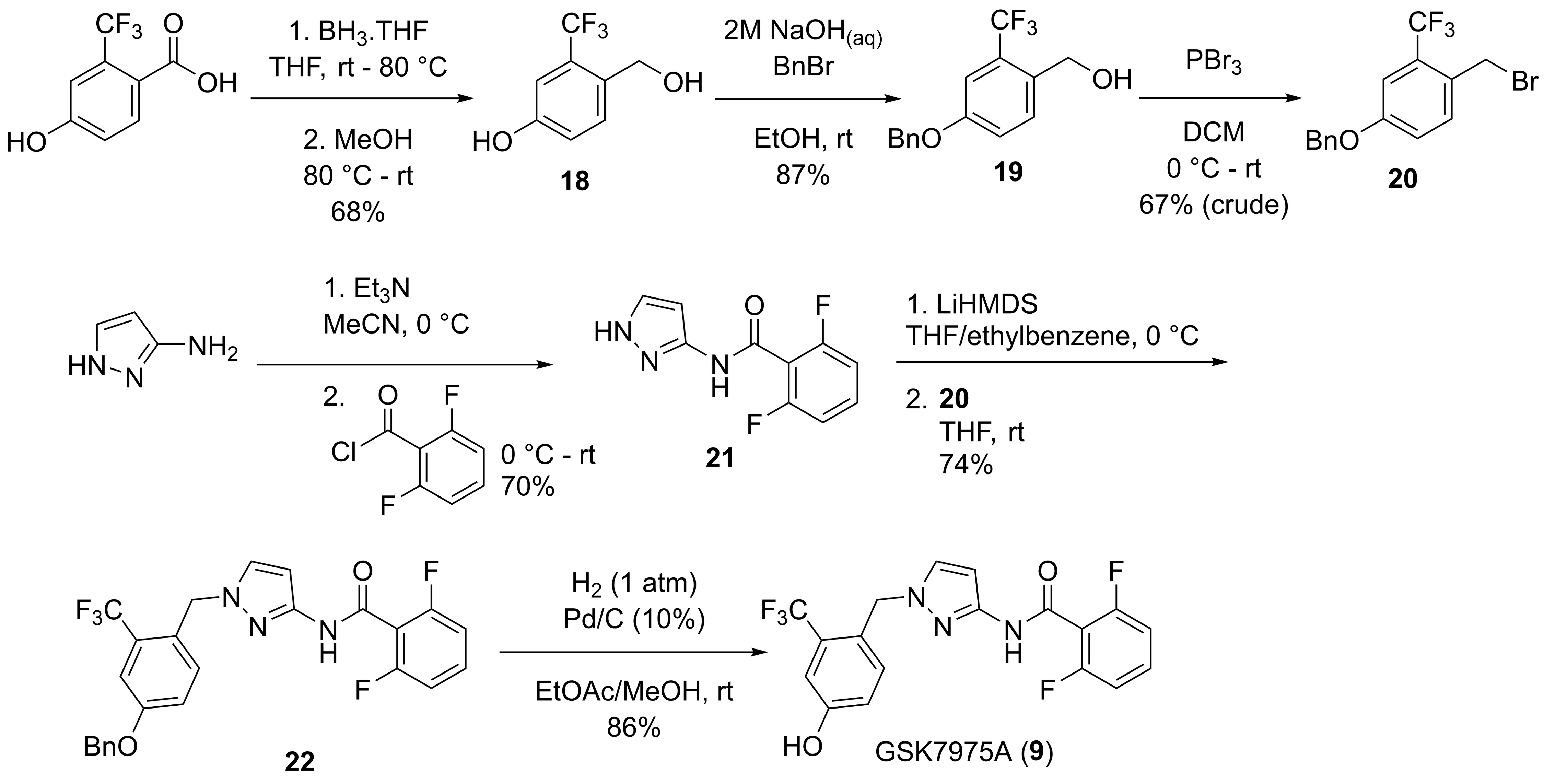

Supplement: S2 Fig — (TIF) [file pone.0296065.s002.tif]

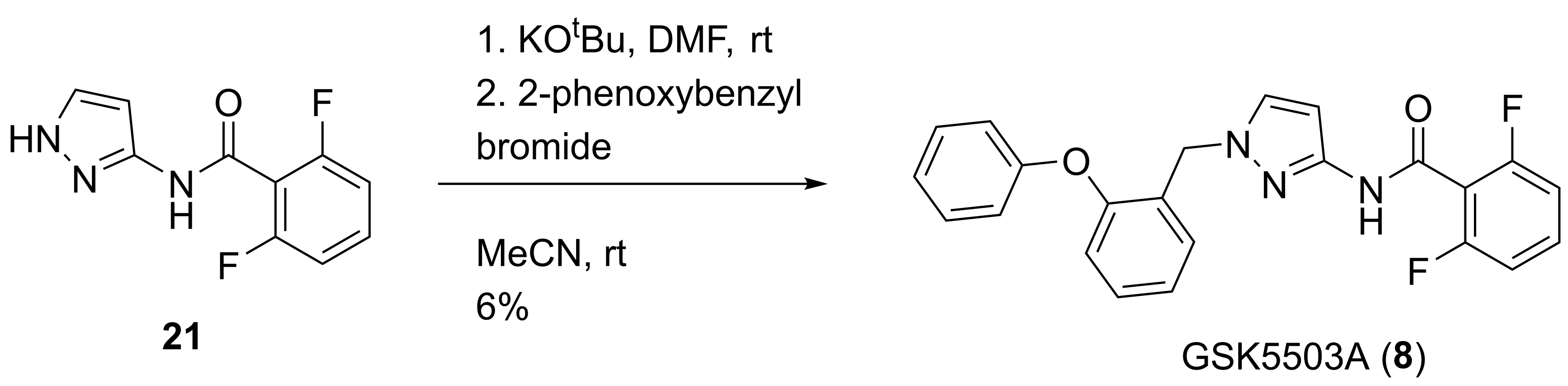

Supplement: S3 Fig — (TIF) [file pone.0296065.s003.tif]

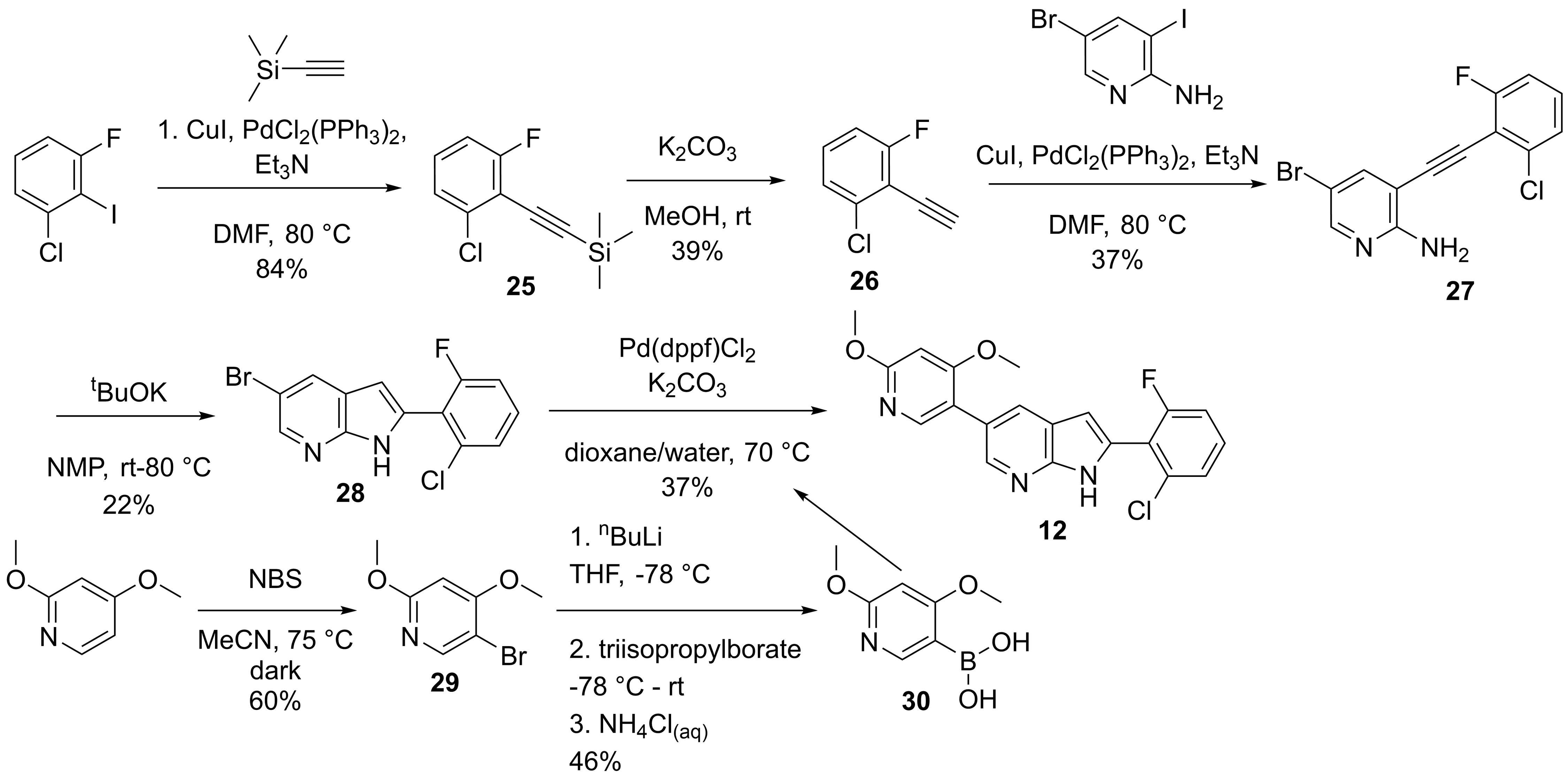

Supplement: S4 Fig — (TIF) [file pone.0296065.s004.tif]

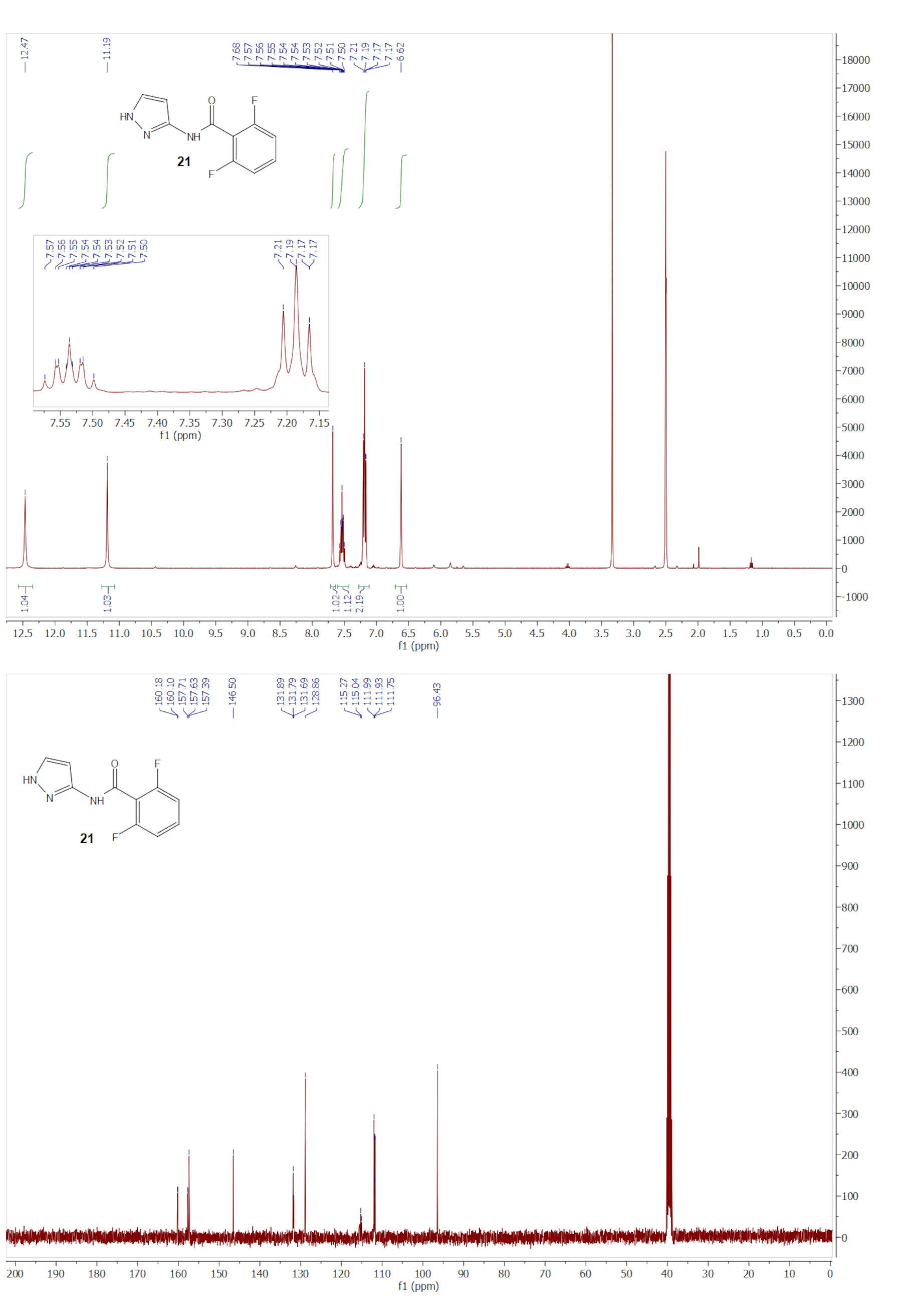

Supplement: S5 Fig — (TIF) [file pone.0296065.s005.tif]

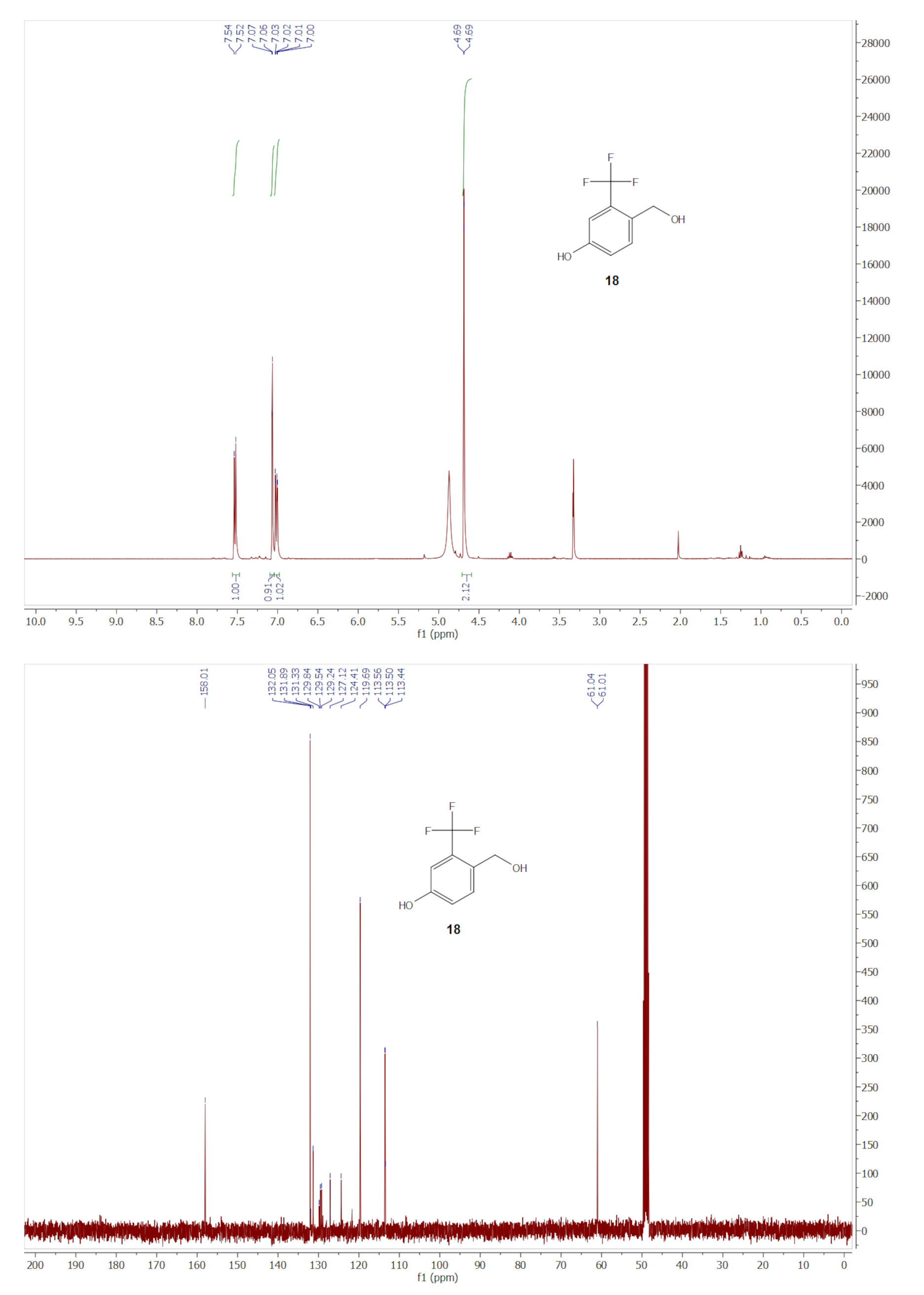

Supplement: S6 Fig — (TIF) [file pone.0296065.s006.tif]

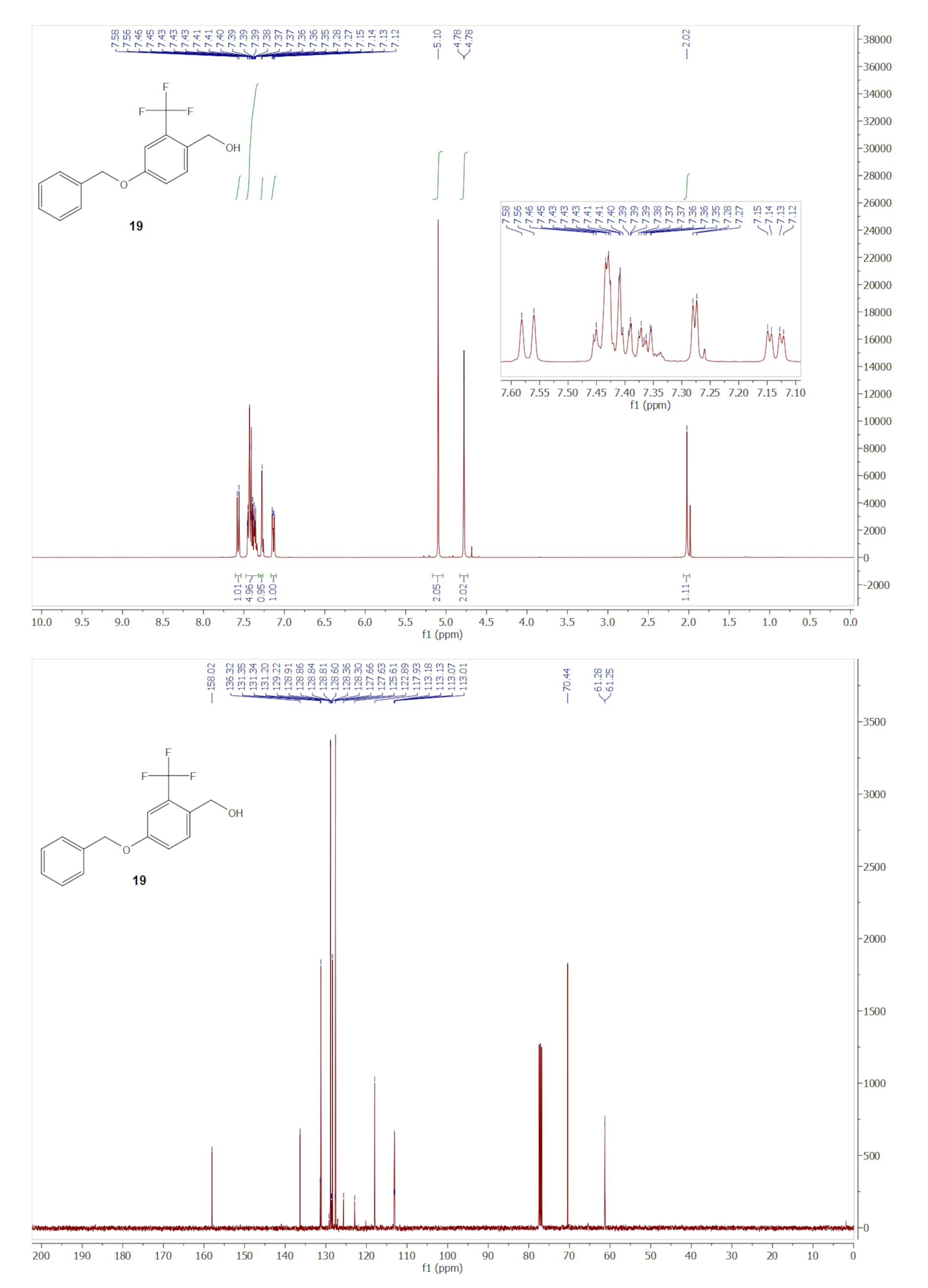

Supplement: S7 Fig — (TIF) [file pone.0296065.s007.tif]

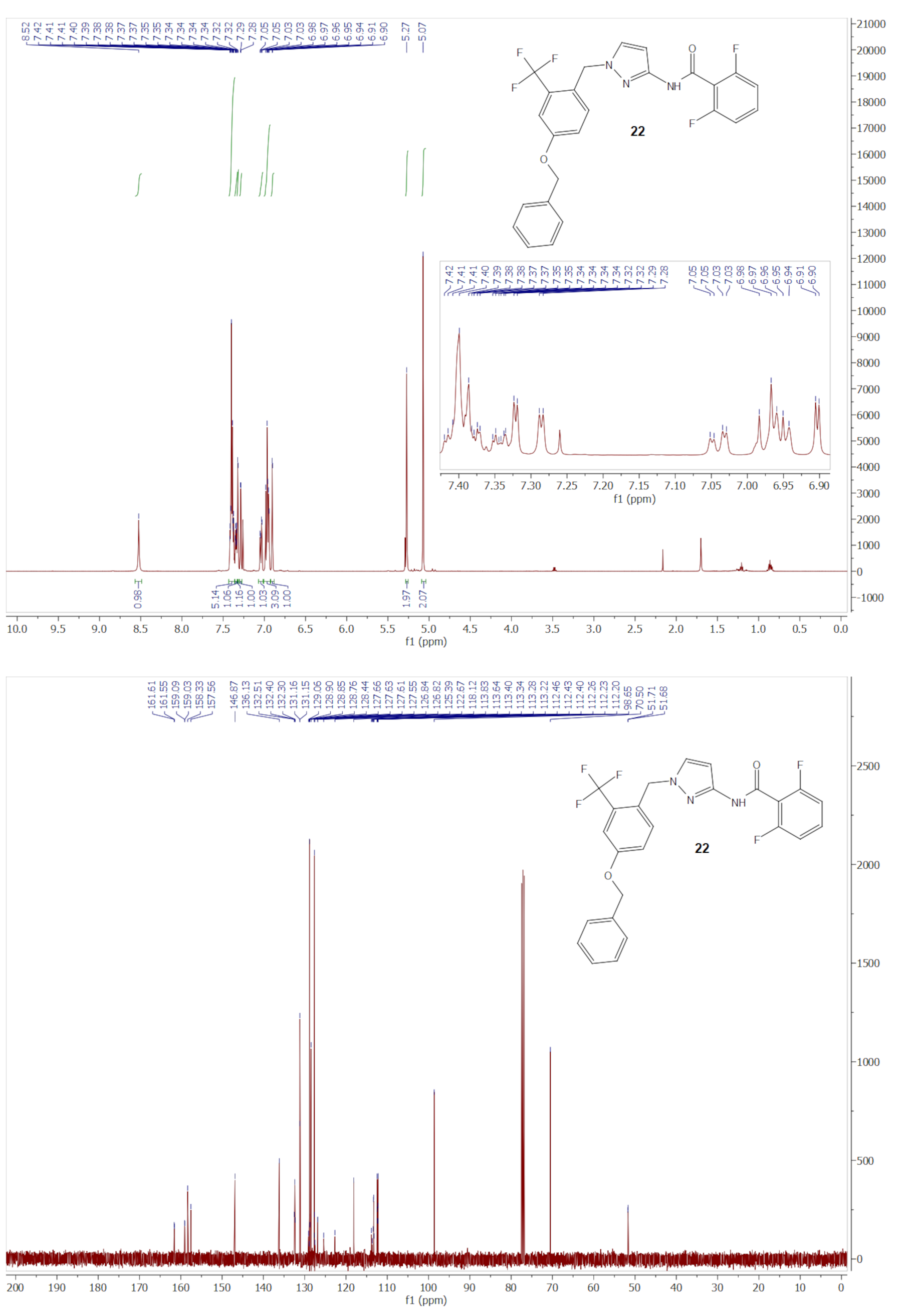

Supplement: S8 Fig — (TIF) [file pone.0296065.s008.tif]

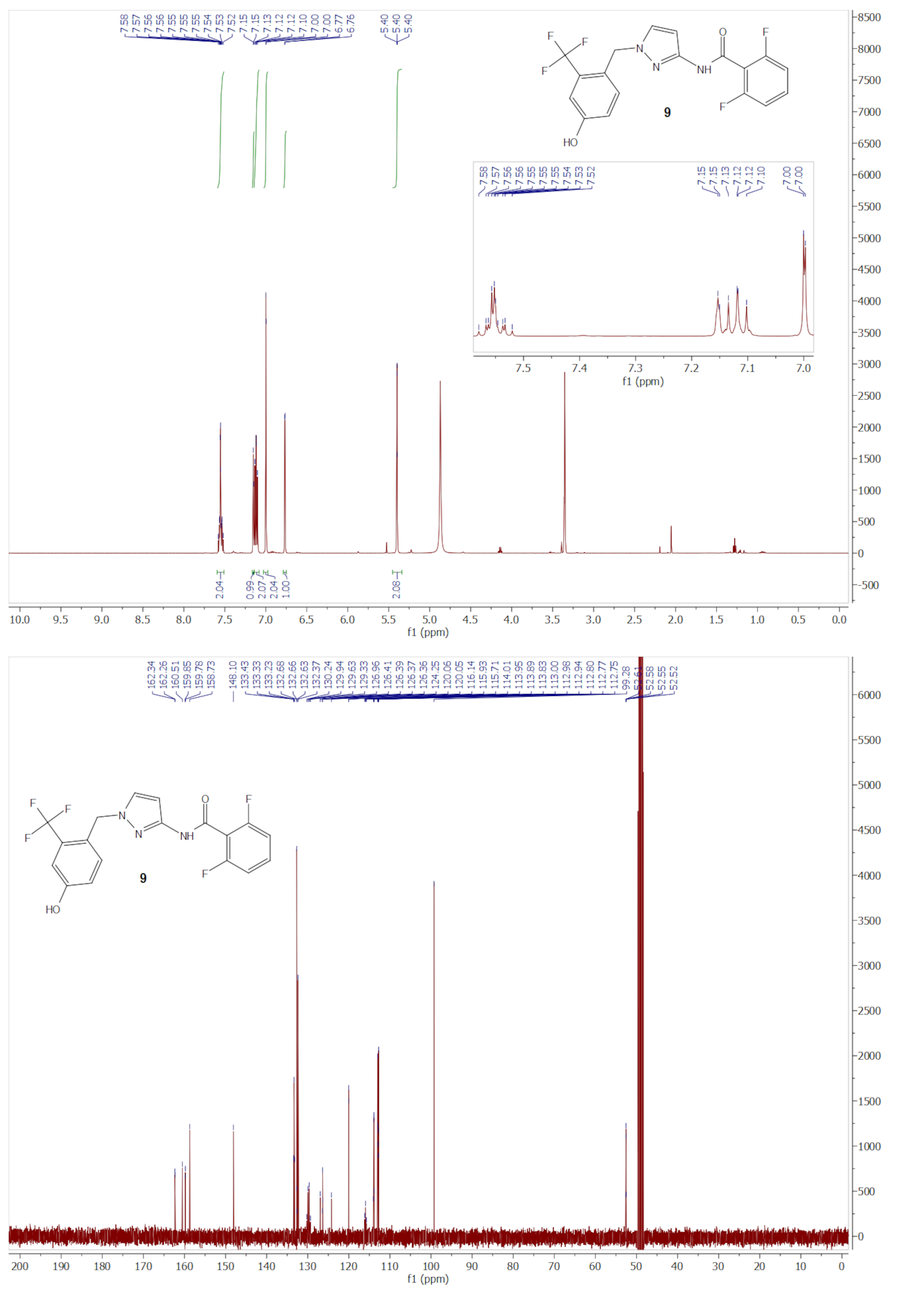

Supplement: S9 Fig — (TIF) [file pone.0296065.s009.tif]

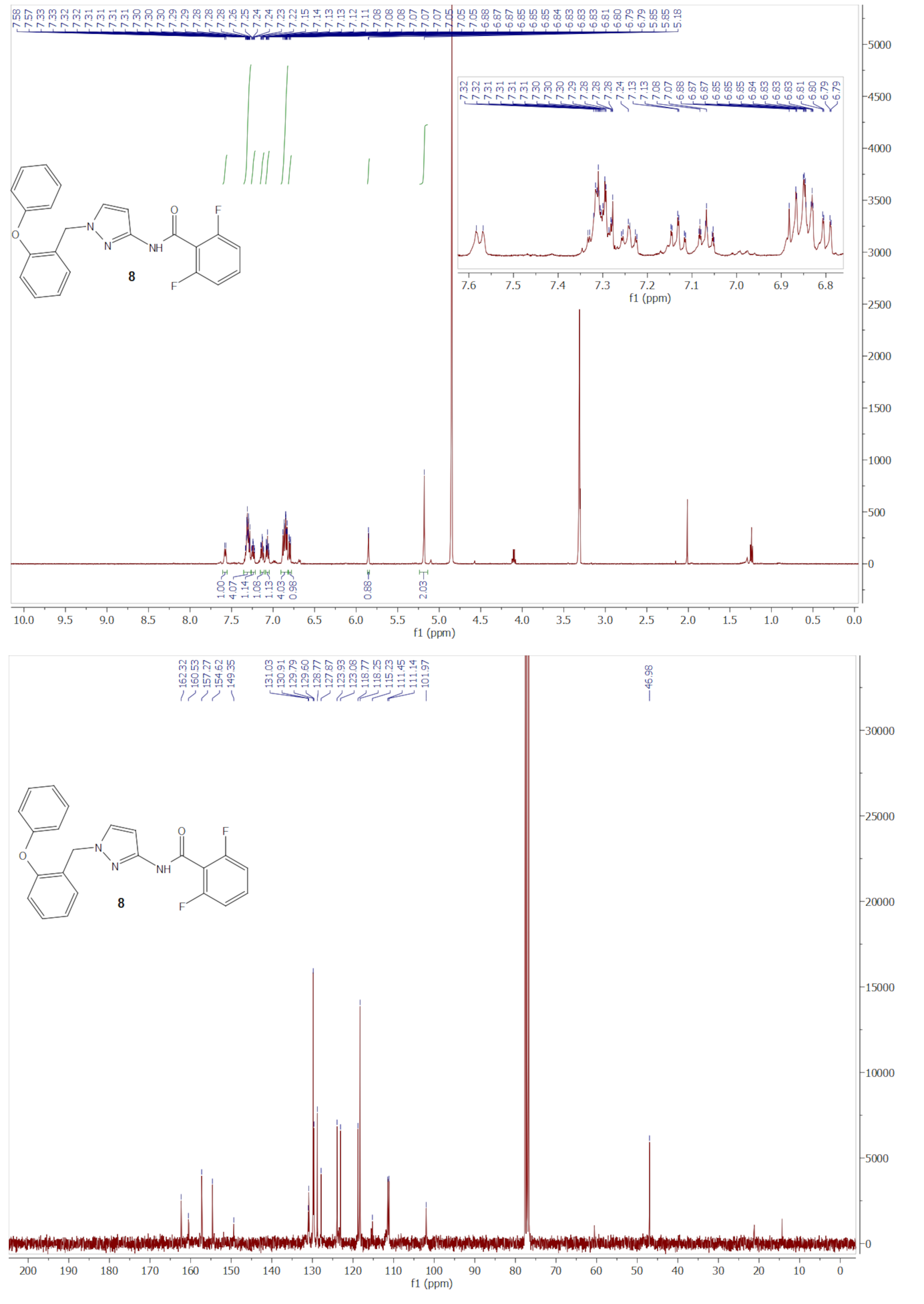

Supplement: S10 Fig — (TIF) [file pone.0296065.s010.tif]

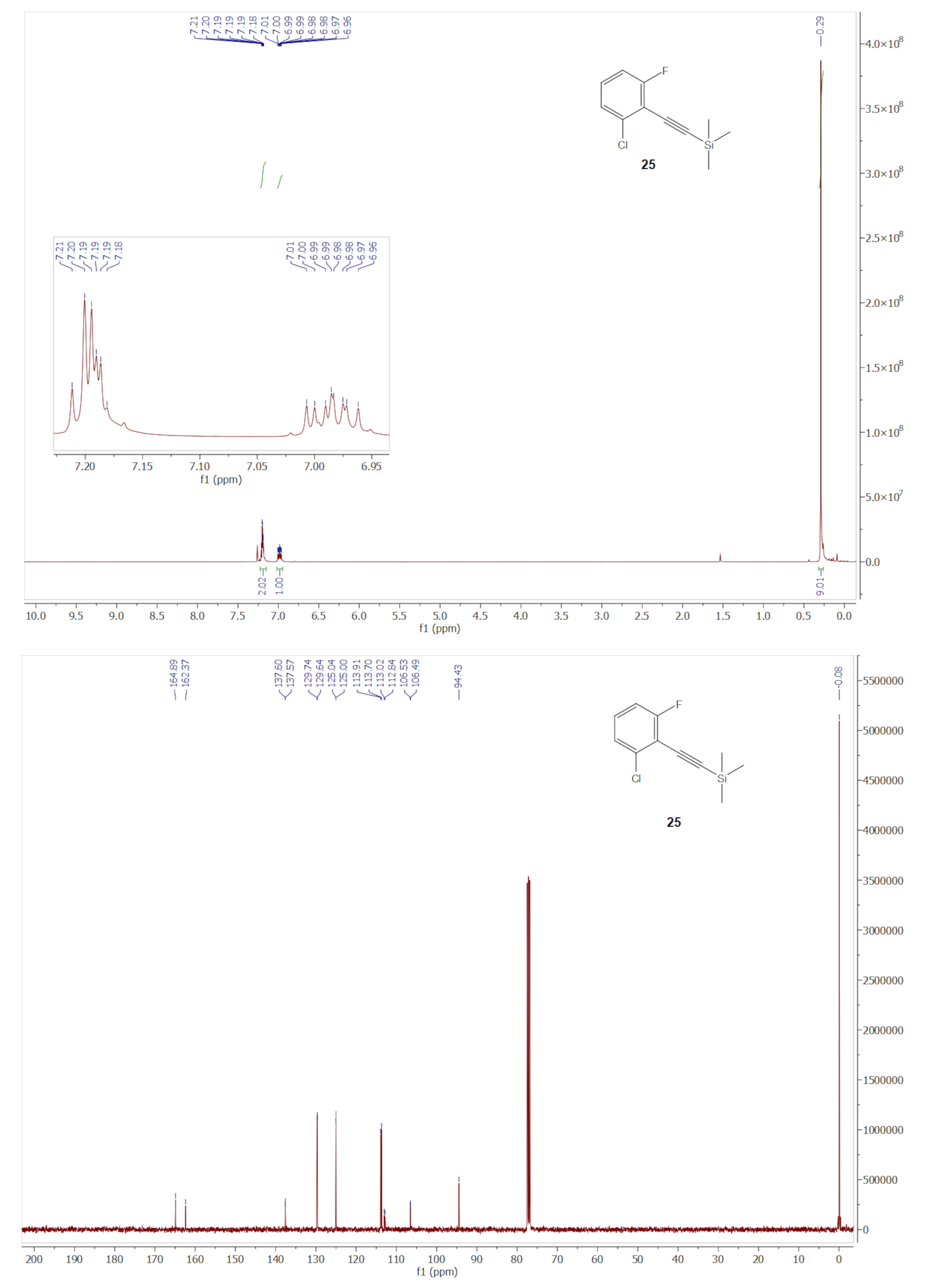

Supplement: S11 Fig — (TIF) [file pone.0296065.s011.tif]

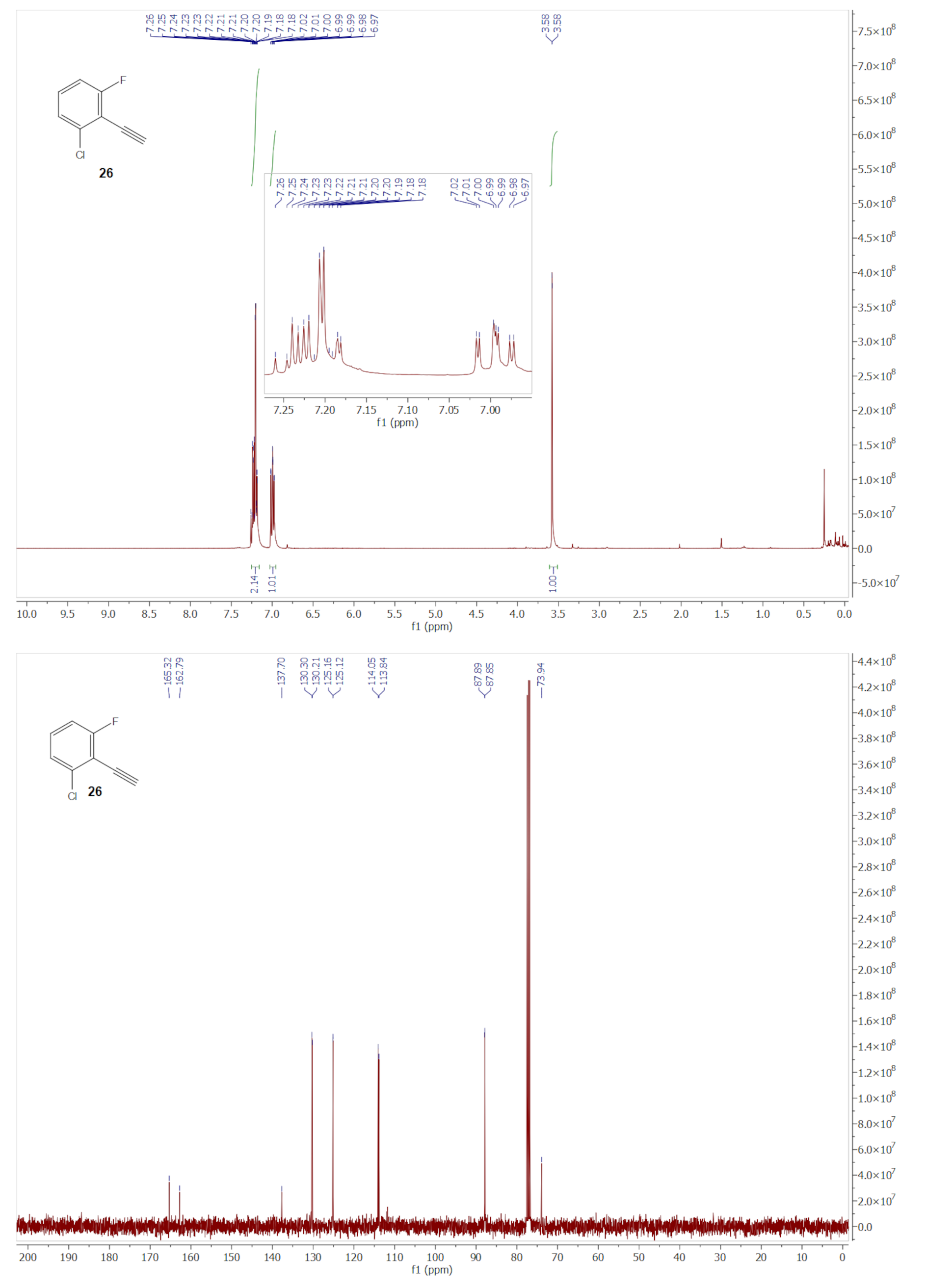

Supplement: S12 Fig — (TIF) [file pone.0296065.s012.tif]

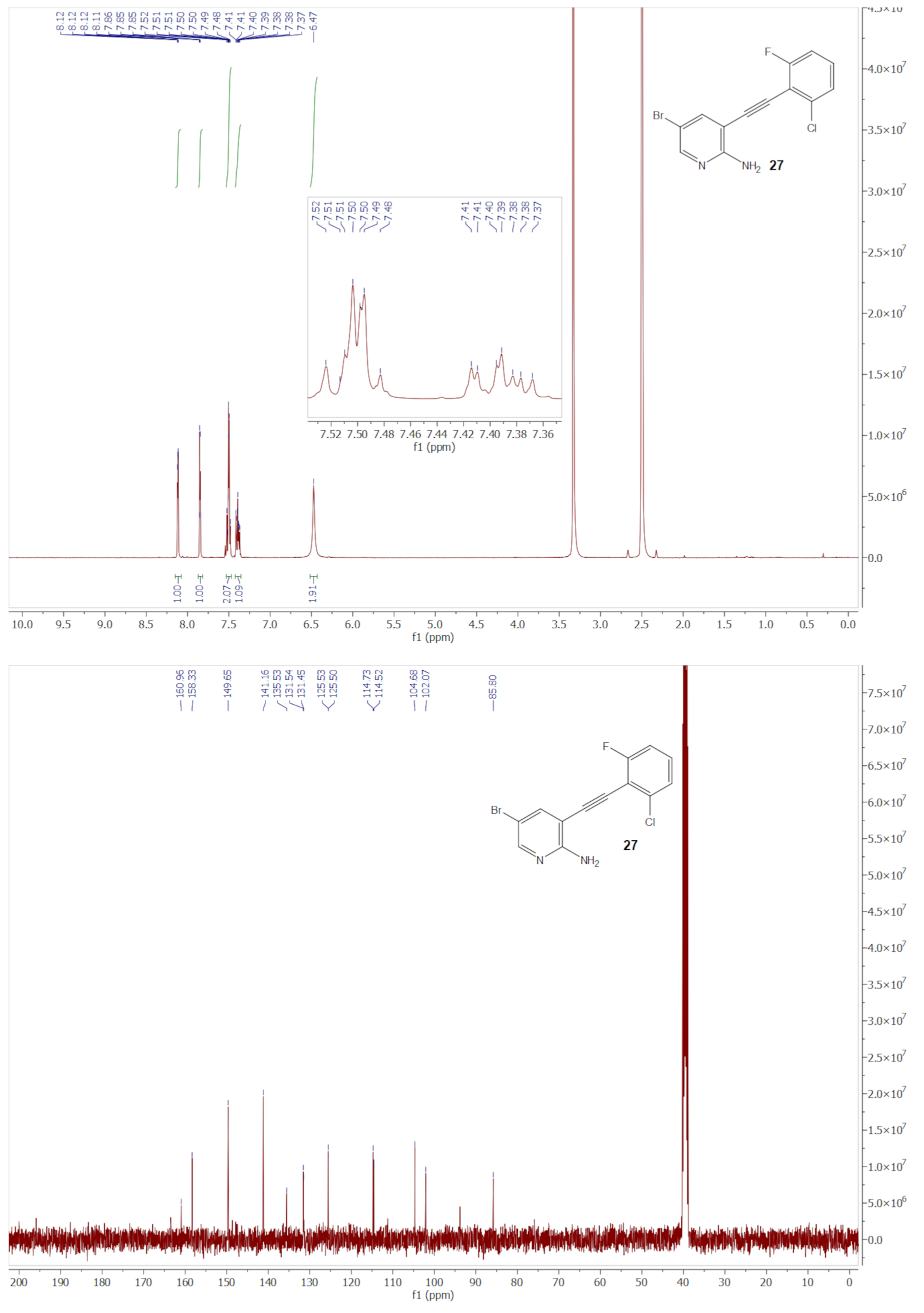

Supplement: S13 Fig — (TIF) [file pone.0296065.s013.tif]

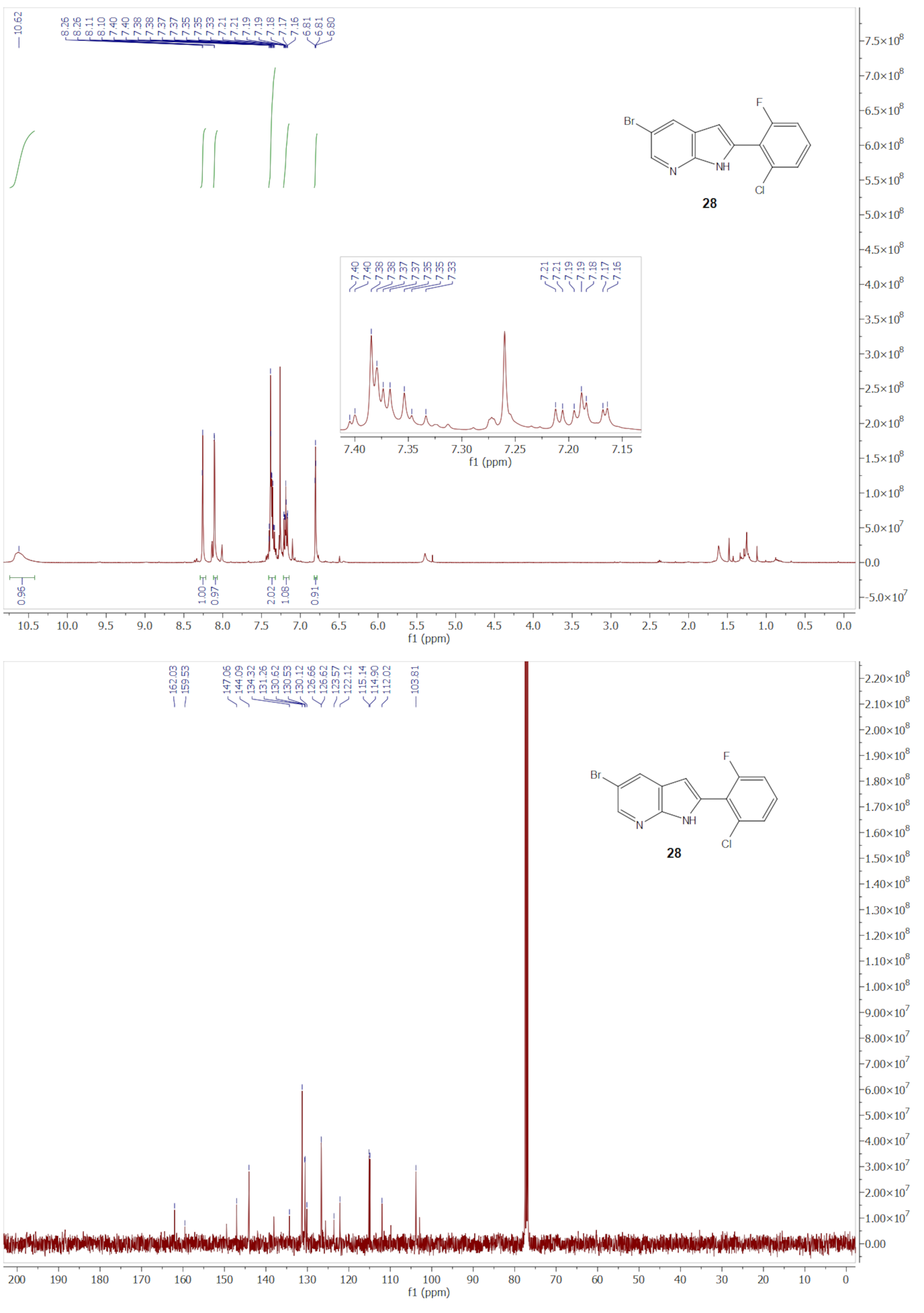

Supplement: S14 Fig — (TIF) [file pone.0296065.s014.tif]

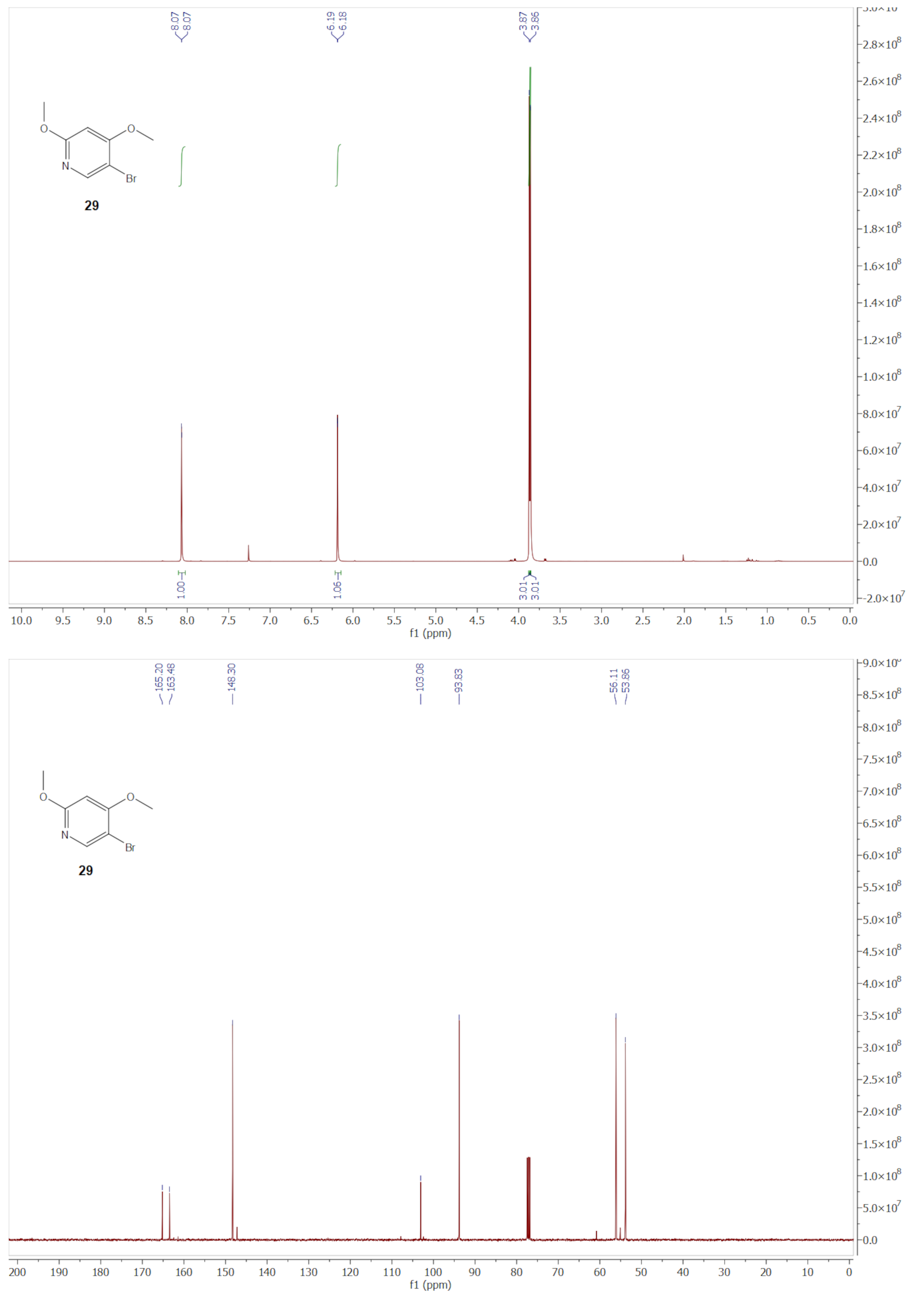

Supplement: S15 Fig — (TIF) [file pone.0296065.s015.tif]

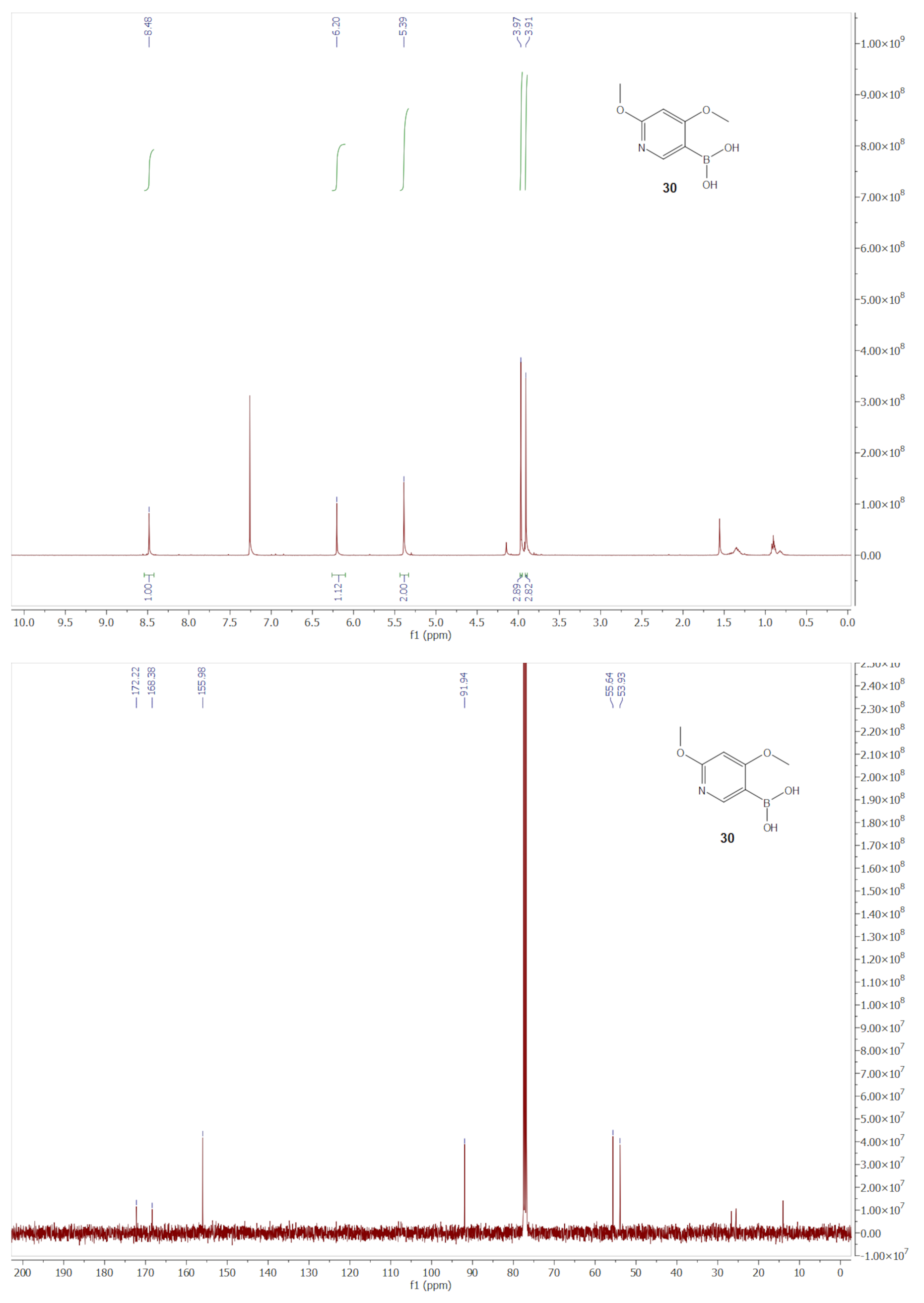

Supplement: S16 Fig — (TIF) [file pone.0296065.s016.tif]

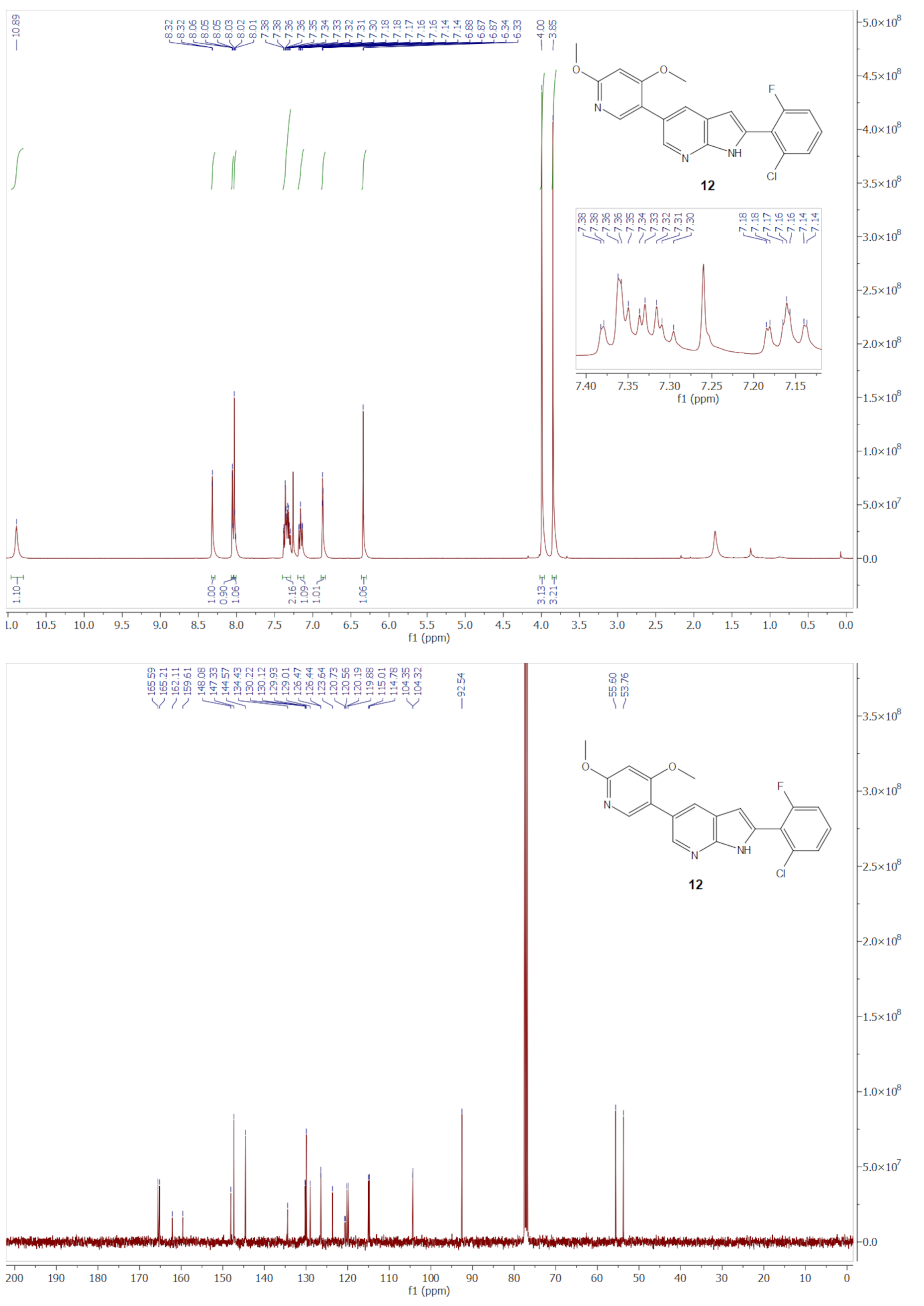

Supplement: S17 Fig — (TIF) [file pone.0296065.s017.tif]

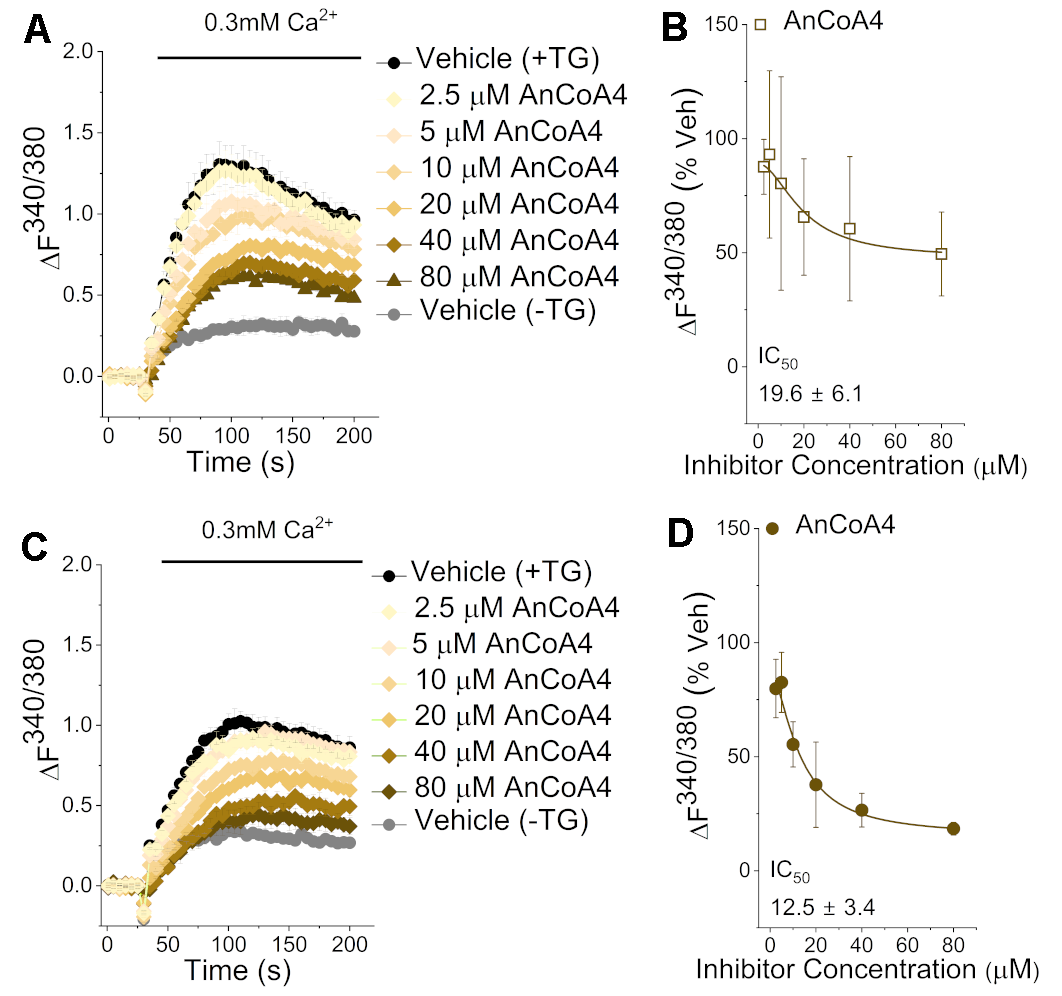

Supplement: S18 Fig — Example fluorescence over time graph (A) and IC50 (B) for AnCoA4 following 30 minute preincubation and example fluorescence over time graph (C) and IC50 (D) for AnCoA4 following 90 minute preincubation. (TIF) [file pone.0296065.s018.tif]

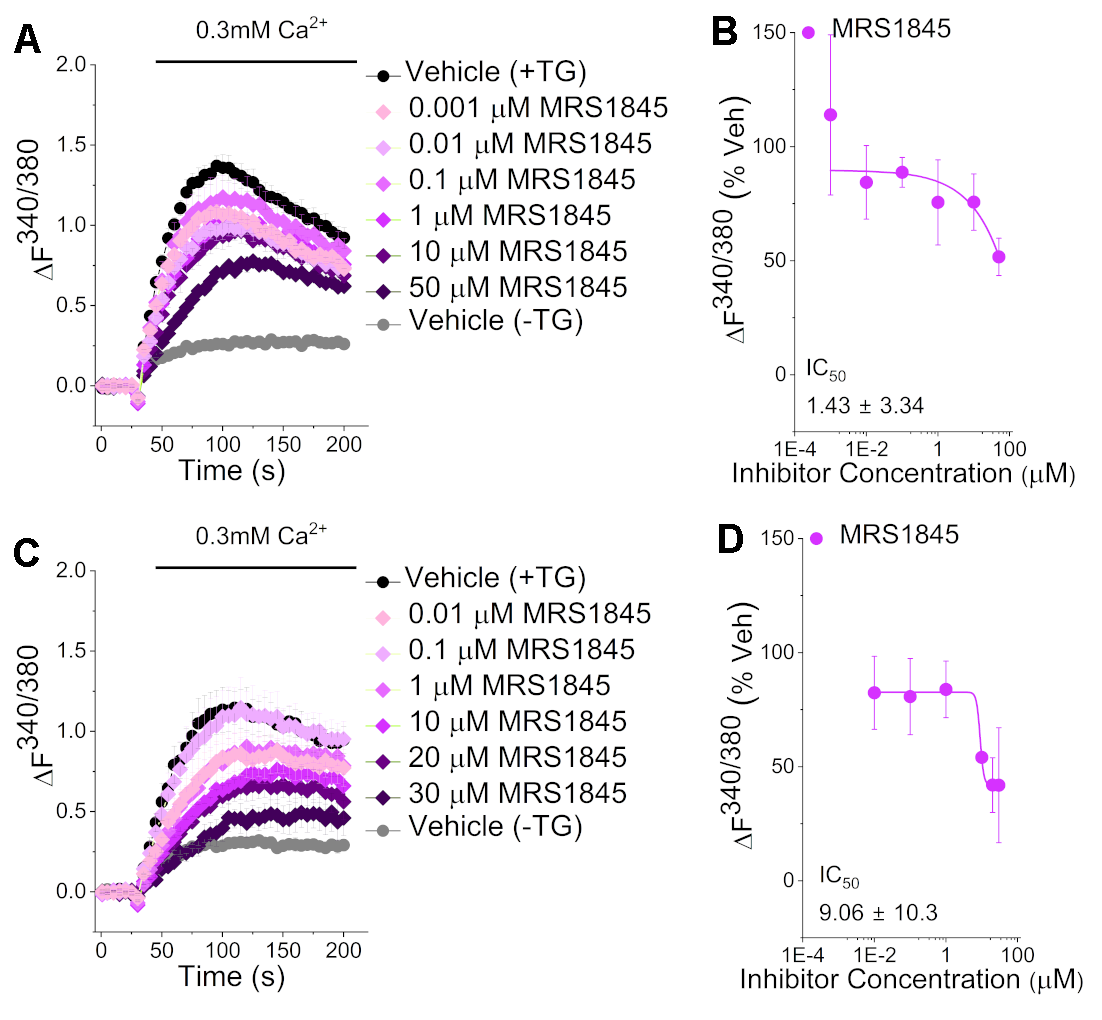

Supplement: S19 Fig — Example fluorescence over time graph (A) and IC50 (B) for MRS1845 following 30 minute preincubation and example fluorescence over time graph (C) and IC50 (D) for MRS1845 following 90 minute preincubation. (TIF) [file pone.0296065.s019.tif]
