# Supplementary material for: Side-by-side comparison of published small molecule inhibitors against thapsigargin-induced store-operated Ca2+ entry in HEK293 cells
Source: PLoS One. 2024 Jan 23;19(1):e0296065. doi: 10.1371/journal.pone.0296065 (PMC10805320; doi:10.1371/journal.pone.0296065)
Supplement: S1 Appendix — (PDF) [file pone.0296065.s020.pdf]

Detailed methods for compound synthesis

All chemicals and solvents were used as supplied. NMR spectra were recorded on either a two-channel Bruker AV3HD NMR spectrometer operating at 9.4 T (400 MHz ^{1}H, 100 MHz ^{13}C) and equipped with a 5 mm BBO probe, a two-channel Bruker AV-NEO NMR spectrometer operating at 11.7 T (500 MHz ^{1}H, 125 MHz ^{13}C) and equipped with a 5 mm DCH cryoprobe or a three-channel Bruker AV operating at 11.7 T (500 MHz ^{1}H, 125 MHz ^{13}C) and equipped with a 5 mm BBO probe. All NMR datasets were acquired at 298 K unless otherwise stated. All ^{1}H and ^{13}C NMR spectra are ^{19}F-coupled. 1H spectra are referenced based on the residual proton in the solvent. Corrected coupling constants (*J*) are reported to the nearest 0.1 Hz for ^{1}H or 1 Hz for ^{13}C, 1H chemical shift to the nearest 0.01 ppm and ^{13}C chemical shift to the nearest 0.1 ppm. NMR data was analysed and coupling constants calculated using TopSpin version 3.0 or 4.0 (Bruker). NMR splitting assigned as: singlet (s), doublet (d), triplet (t), quartet (q), doublet of doublets (dd), triplet of triplets (tt), multiplet (m), broad (br), apparent (app). Infrared spectra were recorded on a Bruker Alpha Platinum ATR FTIR spectrometer and all samples analysed as solids. LCMS was performed on a Thermo Scientific Dionex UltiMate running a gradient of increasing MeCN (5 to 95 %) in water both containing 0.1% formic acid at 1 mL/min, on a short path C18 reverse phase column, detecting compounds with both a diode array detector and a Bruker amaZon speed mass spectrum analyser. Biotage silica column chromatography was carried out using an Isolera Four EXP with Spektra.

2,6-Difluoro-*N*-(1*H*-pyrazol-3-yl)benzamide (21)

To a solution of 3-amino-1*H*-pyrazole (1.836 g, 22.1 mmol) in dry acetonitrile (10 mL) under N_2 at ~0 °C (ice bath) was added triethylamine (6.2 mL, 44.8 mmol) dropwise over 5 minutes. The solution was stirred at 0 °C for 45 minutes, after which a solution of 2,6-difluorobenzoyl chloride (2.8 mL, 22.2 mmol) in acetonitrile (10 mL, kept at ~0 °C) was added dropwise over 45 minutes. The solution was stirred at 0 °C for a further 2.5 hours and at room temperature for a further hour. The reaction mixture was evaporated *in vacuo* and the yellow solid residue partitioned between EtOAc (50 mL) and water (50 mL) and the layers separated. The aqueous phase was extracted with further EtOAc (4 × 50 mL) and the combined organics washed with water (50 mL) and brine (50 mL), dried ($MgSO_4$) and evaporated *in vacuo* to give a light yellow solid. This was suspended in DCM (20 mL), the suspension filtered and the solid washed with further DCM (2 × 10 mL) and the resulting white solid dried at the filter to give the *title compound* as a white powder (3.47 g, 15.6 mmol, 70% yield). ^{1}H NMR δ_H/ppm (400 MHz, DMSO-D_6): 12.47 (1H, br s, pyrazolyl NH), 11.19 (1H, br s, N*H*C(O)), 7.69 (1H, br s, pyrazolyl H-5), 7.54 (1H, tt, *J* 8.3, 6.8 Hz, benzamido H-4), 7.19 (2H, app t, benzamido H-3), 6.62 (1H, br s, pyrazolyl H-4); ^{13}C NMR δ_C/ppm (100 MHz, DMSO-D_6): 159.4 (dd, *J* 258, 8 Hz, benzamido C-2), 157.9 (NHC(O)), 147.0 (pyrazolyl C-3), 132.3 (t, *J* 10 Hz, benzamido C-4), 129.3 (pyrazolyl C-5), 115.7 (t, *J* 21 Hz, benzamido C-1), 112.3 (dd, *J* 19 Hz benzamido C-3), 96.9 (pyrazolyl C-4); $_{max}$/cm^{-1}: 3216 (N-H), 3139 (N-H), 1662 (C=O), 1587, 1462, 1008; HRMS *m*/*z* found: $[C_{10}H_7F_2NaN_3O]^+$ = 246.0449; $[C_{10}H_7F_2NaN_3O]^+$ requires 246.0455. NMR spectra presented in S6 Fig.

3-Trifluoromethyl-4-(hydroxymethyl)phenol (18)

To a solution of 2-trifluoromethyl-4-hydroxybenzoic acid (1.01 g, 4.90 mmol) in dry THF (10 mL) under N_2 was added BH_3.THF (1M in THF, 10.0 mL, 10.0 mmol) dropwise. The colourless solution was heated to 80 °C and stirred for 3 hours. The solution was allowed to cool to room temperature, quenched by dropwise addition of MeOH (10 mL), heated to 80 °C and stirred for a further 30 minutes. It was then cooled to room temperature, the volatiles evaporated *in vacuo*, the residue azeotroped with MeOH (20 mL) and the volatiles evaporated *in vacuo* to give a viscous orange oil. This was purified by column chromatography on silica gel using a gradient of EtOAc/petroleum ether (0-30%) to give the *title compound* as a white solid (637 mg, 3.32 mmol, 68% yield). ^{1}H NMR δ_H/ppm (500 MHz, methanol-D_4): 7.53 (1H, d, *J* 8.4 Hz, phenyl H-5), 7.07 (1H, d, *J* 2.5 Hz, phenyl H-2), 7.02 (1H, dd, *J* 8.4, 2.5 Hz, phenyl H-6), 4.69 (2H, s, C*H*$_2$OH); ^{13}C NMR δ_C/ppm (100 MHz, methanol-D_4): 156.6 (phenyl C-1), 130.7 (phenyl C-5), 129.9 (phenyl C-4), 128.0 (q, *J* 31 Hz, phenyl C-3), 124.4 (q, *J* 273 Hz, *C*F_3), 118.3 (phenyl C-6), 112.1 (q, *J* 6 Hz, phenyl C-2), 59.6 (q, *J* 3 Hz, CH_2OH); $_{max}$/cm^{-1}: 3385 (phenol O-H), 3106 (aliphatic O-H), 1318, 1228, 1105 (C-F), 1021 (aliphatic C-O); Compound does not ionise to give correct product mass on HRMS. NMR spectra presented in S7 Fig.

[4-(Benzyloxy)-2-(trifluoromethyl)phenyl]methanol (19)

To a stirring solution of 3-trifluoromethyl-4-(hydroxymethyl)phenol (**18**) (636 mg, 3.31 mmol) in EtOH (5 mL) was added 2M $NaOH_{(aq)}$ (1.8 mL, 3.6 mmol), followed by benzyl bromide (470 µL, 3.96 mmol). The solution was stirred at room temperature under N_2 for 42 hours, after which EtOH was removed *in vacuo* and EtOAc (10 mL) and water (10 mL) added to the residue. The layers were separated and the aqueous was extracted with further EtOAc (2 × 10 mL) and the combined organics washed with water (10 mL) and brine (10 mL), dried ($MgSO_4$) and evaporated *in vacuo* to give a light yellow oil. This was purified using column chromatography on silica gel using a gradient of EtOAc/petroleum ether (0-50%) to give the *title compound* as a white waxy solid (814 mg, 2.89 mmol, 87% yield). ^{1}H NMR δ_H/ppm (400 MHz, $CDCl_3$): 7.57 (1H, d, *J* 8.6 Hz, phenyl H-6), 7.45-7.34 (5H, m, benzyl), 7.28 (1H, d, *J* 2.6 Hz, phenyl H-3), 7.14 (1H, dd, *J* 8.6, 2.6 Hz, phenyl H-5), 5.10 (2H, s, C*H*$_2$OPh), 4.78 (2H, s, C*H*$_2$OH), 2.02 (1H, br s, OH); ^{13}C NMR δ_C/ppm (100 MHz, $CDCl_3$): 157.9 (phenyl C-4), 136.2 (benzyl C-1), 131.2 (phenyl C-1), 131.1 (phenyl C-6), 128.7 (benzyl C-2 or C-3), 128.6 (q, *J* 31 Hz, phenyl C-2), 128.3 (benzyl C-4), 127.5 (benzyl C-2 or C-3), 124.2 (q, *J* 274 Hz, *C*F_3), 117.8 (phenyl C-5), 113.0 (q, *J* 6 Hz, phenyl C-3), 70.4 (CH_2OPh), 61.2 (app d, *J* 4 Hz, CH_2OH); $_{max}$/cm^{-1}: 3313 (O-H), 1287, 1241, 1150, 1105 (C-F), 1023 (aliphatic C-O); HRMS *m/z* found: $[C_{15}H_{13}F_3NaO_2]^+$ = 305.0756; $[C_{15}H_{13}F_3NaO_2]^+$ requires 305.0760. NMR spectra presented in S8 Fig.

***N*-(1-{[4-(Benzyloxy)-2-(trifluoromethyl)phenyl]methyl}-1*H*-pyrazol-3-yl)-2,6-difluorobenzamide (22)**

To a suspension of [4-(benzyloxy)-2-(trifluoromethyl)phenyl]methanol (**19**) (749 mg, 2.66 mmol) in dry DCM (8 mL) under N_2 at ~0 °C was added PBr_3 (250 µL, 2.66 mmol) dropwise. The yellow solution was allowed to warm to room temperature and stirred for 4 hours, after which it was poured onto ice water (~10 mL) and saturated $NaHCO_{3(aq)}$ (20 mL). DCM (40 mL) was added and the layers separated. The aqueous was extracted with further DCM (2 × 40 mL) and the combined organics dried ($MgSO_4$) and evaporated *in vacuo* to give crude 4-

(benzyloxy)-1-(bromomethyl)-2-(trifluoromethyl)benzene (**20**) as a light yellow oil which formed crystals upon standing (612 mg, 1.78 mmol, 67% yield).

To a solution of 2,6-difluoro-*N*-(1*H*-pyrazol-3-yl)benzamide (**21**) (312 mg, 1.40 mmol) in dry THF (4.0 mL) at 0 °C was added LiHMDS (1.0 M in THF/ethylbenzene, 1.40 mL, 1.40 mmol) and the yellow solution stirred at 0 °C under N_2 for 30 minutes. To the solution was added a solution of the crude 4-(benzyloxy)-1-(bromomethyl)-2-(trifluoromethyl)benzene (**20**) (612 mg, 1.78 mmol) in THF (4.0 mL) dropwise and the resulting yellow suspension allowed to warm to room temperature and stirred for 21 hours under N_2. Volatiles were removed *in vacuo* and the residue partitioned between EtOAc (20 mL) and saturated $NaHCO_{3(aq)}$ (20 mL) and the layers separated. The aqueous was extracted with further EtOAc (3 × 20 mL) and the combined organics washed with $NaHCO_{3(aq)}$ (20 mL), and brine (20 mL), dried ($MgSO_4$) and evaporated *in vacuo* to give a viscous yellow oil. This was purified by column chromatography on silica gel using a gradient of EtOAc/petroleum ether (0-40%) to give the *title compound* as a glassy colourless solid (502 mg, 1.03 mmol, 74% yield). ^{1}H NMR δ_H/ppm (500 MHz, $CDCl_3$): 8.53 (1H, br s, NH), 7.43-7.37 (5H, m, benzamido H-4, benzyl H-2, benzyl H-3), 7.36-7.34 (1H, m, benzyl H-4), 7.33 (1H, d, *J* 2.3 Hz, pyrazolyl H-5), 7.29 (1H, d, *J* 2.6 Hz, phenyl H-3), 7.04 (1H, dd, *J* 8.6, 2.6 Hz, phenyl H-5), 7.00-6.94 (3H, m, phenyl H-6, benzamido H-3), 6.91 (1H, d, *J* 2.3 Hz, pyrazolyl H-4), 5.23 (PhCH_2), 5.08 (OCH_2Ph); ^{13}C NMR δ_C/ppm (100 MHz, $CDCl_3$): 160.2 (dd, *J* 254, 6 Hz, benzamido C-2), 158.2 (NH*C*(O)), 157.4 (phenyl C-4), 146.7 (pyrazolyl C-3), 136.0 (benzyl C-1), 132.3 (t, *J* 10 Hz, benzamido C-4), 131.0 (pyrazolyl C-5, phenyl C-6), 128.8 (q, *J* 31 Hz, phenyl C-2), 128.7 (benzyl C-2 or C-3), 128.3 (benzyl C-4), 127.5 (benzyl C-2 or C-3), 126.7 (phenyl C-1), 123.9 (q, *J* 274 Hz, *C*F_3), 118.0 (phenyl C-5), 113.7 (t, *J* 19 Hz, benzamido C-1), 113.2 (q, *J* 6 Hz, phenyl C-3), 112.2 (d, *J* 26 Hz, benzamido C-3), 98.5 (pyrazolyl C-4), 70.4 (OCH_2Ph), 51.6 ($PhCH_2$); $_{max}$/cm^{-1}: 3233 (N-H), 1683 (C=O), 1575, 1466, 1312, 1114, 1003; HRMS *m/z* found: $[C_{25}H_{18}F_5N_3NaO_2]^+$ = 510.1208; $[C_{25}H_{18}F_5N_3NaO_2]^+$ requires 510.1211. NMR spectra presented in S9 Fig.

2,6-Difluoro-*N*-(1-{[4-hydroxy-2-(trifluoromethyl)phenyl]methyl}-1*H*-pyrazol-3-yl)benzamide (GSK7975A, 9)

To a solution of *N*-(1-{[4-(benzyloxy)-2-(trifluoromethyl)phenyl]methyl}-1*H*-pyrazol-3-yl)-2,6-difluorobenzamide (**23**) (415 mg, 0.92 mmol) in EtOAc (4 mL) and MeOH (2 mL) under N_2 was added Pd/C (10%, wetted) (43 mg). The flask was evacuated and filled with an H_2 atmosphere and the reaction mixture stirred at room temperature for 10 hours. The reaction mixture was filtered through celite twice and the filtrate evaporated *in vacuo* to give a grey gum. This was purified by column chromatography on silica gel using a gradient of EtOAc/petroleum ether (0-60%) to give the *title compound* as a colourless glassy solid (336 mg, 0.85 mmol, 86% yield). ^{1}H NMR δ_H/ppm (500 MHz, methanol-D_4): 7.55 (1H, d, *J* 2.4 Hz, pyrazolyl H-5), 7.55 (1H, tt, *J* 8.5, 6.4 Hz, benzamido H-4), 7.15 (1H, br s, phenyl H-3), 7.12 (2H, dd, *J* 8.5, 7.9 Hz, benzamido H-3), 7.00 (2H, app d, phenyl H-5, H-6), 6.77 (1H, d, *J* 2.4 Hz, pyrazolyl H-4), 5.40 (2H, s, CH_2); ^{13}C NMR δ_C/ppm (100 MHz, methanol-D_4): 161.1 (dd, *J* 250, 7 Hz, benzamido C-2), 160.5 (*C*(O)NH), 158.7 (phenyl C-4), 148.1 (pyrazolyl C-3), 133.3 (t, *J* 10 Hz, benzamido C-4), 131.6 (pyrazolyl C-5), 130.9 (phenyl C-6), 129.8 (q, *J* 30 Hz, phenyl C-2), 126.4 (q, *J* 2 Hz, phenyl C-1), 125.7 (q, *J* 273 Hz, *C*F_3), 119.2 (phenyl C-5), 113.9 (q, *J* 6 Hz, phenyl C-3), 115.9 (t, *J* 22 Hz,

benzamido C-1), 112.9 (dd, *J* 18, 4 Hz, benzamido C-3), 99.3 (pyrazolyl C-4), 52.6 (q, *J* 3 Hz, $PhCH_2$); $_{max}$/cm^{-1}: 3248 (N-H), 2874 (O-H), 1660 (C=O), 1466, 1320, 1112; HRMS *m/z* found: $[C_{18}H_{12}F_5N_3NaO_2]^+$ = 420.0742; $[C_{18}H_{12}F_5N_3NaO_2]^+$ requires 420.0742. NMR spectra presented in S10 Fig.

2,6-Difluoro-*N*-{1-[(2-phenoxyphenyl)methyl]-1*H*-pyrazol-3-yl}benzamide (GSK5503A, 8)
A solution of 2,6-difluoro-*N*-(1*H*-pyrazol-3-yl)benzamide (210 mg, 0.95 mmol) in DMF (1 mL) was treated with KO^tBu (110 mg, 0.95 mmol) and allowed to stir at room temperature for 10 minutes. A solution of 2-phenoxybenzyl bromide (250 mg, 0.95 mmol) in acetonitrile (0.5 mL) was added and the solution was continue stirred for 16 hours at room temperature. The solvent was removed under pressure, dissolved with water (6 mL) and extracted with EtOAc (2 × 6 mL). The organic layers were combined, dried (Na_2SO_4), filtered and removed to dryness. The crude was purified by column chromatography on silica gel using a gradient of EtOAc/petroleum ether (0-100%) to give the *title compound* as a colourless solid (25 mg, 0.062 mmol, 6% yield). ^{1}H NMR δ_H/ppm (500 MHz, methanol-D_4): 7.58 (1H, app d, *J* 7.4 Hz, benzamido H-4), 7.35-7.26 (4H, m, phenoxy H-3, phenyl H-4 , pyrazolyl H-5), 7.24 (1H, dd, *J* 7.6, 1.8 Hz, phenyl H-6) 7.13 (1H, ddd, *J* 7.6, 7.5, 1.2 Hz, phenyl H-5), 7.07 (1H, tt, *J* 7.4, 1.1 Hz, phenoxy H-4) 6.89-6.83 (4H, m, phenoxy H-2, benzamido H-3), 6.80 (1H, dd, *J* 8.2, 1.2 Hz, phenyl H-3), 5.85 (1H, d, *J* 2.6 Hz, pyrazolyl H-4), 5.18 (2H, s, $PhCH_2$); ^{13}C NMR δ_C/ppm (125 MHz, $CDCl_3$): 162.2 (C=O), 158.7 (dd, *J* = 250, 7 Hz, benzamido C-2), 154.5 (phenyl C-2, phenoxy C-1), 149.2 (pyrazolyl C-3), 130.8 (t, *J* = 10 Hz, benzamido C-4), 129.5 (phenoxy C-2), 129.7 (pyrazolyl C-5), 127.8 (phenyl C-4), 128.7 (phenyl C-3), 123.8 (phenyl C-5), 123.0 (phenoxy C-3), 118.7 (phenoxy C-4), 118.1 (phenyl C-6), 115.1 (t, *J* 19 Hz, benzamido C-1), 111.2 (dd, *J* 19, 6 Hz, benzamido C-3), 101.9 (pyrazolyl C-4), 46.9 ($PhCH_2$); $_{max}$/cm^{-1}: 3318 (N-H), 1640 (C=O), 1625 (C=C aromatic); HRMS *m/z* found: $[C_{23}H_{18}F_2N_3O_2]^+$ = 406.1362; $[C_{23}H_{18}F_2N_3O_2]^+$ requires 406.1358. NMR spectra presented in S11 Fig.

[(2-Chloro-6-fluorophenyl)ethynyl](trimethyl)silane (25)
A solution of 1-chloro-3-fluoro-2-iodobenzene (2.974 g, 11.7 mmol) and Et_3N (4.9 mL, 35.9 mmol) in DMF (5 mL) was degassed with N_2 for 30 minutes, after which TMS-acetylene (3.2 mL, 23.2 mmol), CuI (93 mg, 0.49 mmol) and $Pd(PPh_3)_2Cl_2$ (161 mg, 0.23 mmol) were added and the resulting orange suspension heated to 80 °C and stirred under N_2 for 1.5 hours. The reaction mixture was cooled to room temperature and filtered through a pad of celite. The celite was washed with EtOAc (2 × 20 mL) and to the filtrate was added brine and water and the layers separated. The aqueous phase was extracted with further EtOAc (2 × 20 mL), the combined organic layers washed with water and brine (20 mL each) and the organic phase was dried ($MgSO_4$) and evaporated *in vacuo*. Crude (viscous black oil) was purified by column chromatography on silica gel using neat petroleum ether to give the *title compound* as an orange oil (2.217 g, 9.81 mmol, 84% yield). ^{1}H NMR δ_H/ppm (400 MHz, $CDCl_3$): 7.21-7.17 (2H, m, phenyl H-3, H-4), 7.00-6.95 (1H, m, phenyl H-5), 0.29 (9H, s, $Si(CH_3)_3$); ^{13}C NMR δ_C/ppm (100 MHz, $CDCl_3$): 163.7 (d, *J* 254 Hz, phenyl C-6), 137.6 (phenyl C-2), 129.8 (d, *J* 9 Hz, phenyl C-4), 125.1 (d, *J* 3 Hz, phenyl C-3), 113.9 (d, *J* 21 Hz, phenyl C-5), 113.0 (d, *J* 19 Hz, phenyl C-1), 106.6 (d, *J* 4 Hz, C≡*C*-Si), 94.5 (*C*≡C-Si), 0.0 ($Si(CH_3)_3$); $_{max}$/cm^{-1}: 2166 (C≡C), 1654, 1460, 1447,

1251 (C-Si), 842 (C-Si); Compound does not ionise to give correct product mass on HRMS. NMR spectra presented in S12 Fig.

1-Chloro-2-ethynyl-3-fluorobenzene (26)

To a solution of [(2-chloro-6-fluorophenyl)ethynyl](trimethyl)silane (**25**) (2.217 g, 9.81 mmol) in MeOH (15 mL) was added K_2CO_3 (3.098 g, 22.4 mmol) and the yellow suspension stirred at room temperature for 1 hour. Volatiles were evaporated *in vacuo* and the residue resuspended in EtOAc (20 mL) and water (20 mL) and the layers separated. The aqueous phase was extracted with further EtOAc (2 × 20 mL) and the combined organic layers washed with water (20 mL) and brine (20 mL). The aqueous was extracted further with DCM (3 × 20 mL) and the combined organic phases dried ($MgSO_4$) and evaporated *in vacuo* to give the *title compound* as a dark orange oil (582 mg, 3.80 mmol, 39% yield). ^{1}H NMR δ_H/ppm (400 MHz, $CDCl_3$): 7.25-7.18 (2H, m, phenyl H-3, H-4), 6.99 (1H, app td, phenyl H-5), 3.58 (1H, br d, *J* 0.5 Hz, alkenyl C-H); ^{13}C NMR δ_C/ppm (100 MHz, $CDCl_3$): 163.9 (d, *J* 255 Hz, phenyl C-3), 137.6 (phenyl C-1), 130.1 (d, *J* 9 Hz, phenyl C-5), 125.0 (d, *J* 4 Hz, phenyl C-6), 113.8 (d, *J* 21 Hz, phenyl C-4), 111.7 (d, *J* 19 Hz, phenyl C-2), 87.7 (d, *J* 4 Hz, *C*≡CH), 73 (C≡CH); $_{max}$/cm^{-1}: 3300, 2116 (C≡C), 1710, 1601, 1570, 1446, 1248; Compound does not ionise to give correct product mass on HRMS. NMR spectra presented in S13 Fig.

5-Bromo-3-[(2-chloro-6-fluorophenyl)ethynyl]pyridin-2-amine (27)

A solution of 1-chloro-2-ethynyl-3-fluorobenzene (**26**) (582 mg, 3.80 mmol), 2-amino-5-bromo-3-iodopyridine (1.097 g, 3.67 mmol) and Et_3N (1.6 mL, 11.6 mmol) in DMF (3 mL) was degassed with N_2 for 30 minutes, after which CuI (33 mg, 0.17 mmol) and $Pd(PPh_3)_2Cl_2$ (52 mg, 0.075 mmol) were added and the resulting orange suspension heated to 80 °C and stirred under N_2 for 4.5 hours. The reaction mixture was cooled to room temperature and filtered through a pad of celite. The celite was washed with EtOAc (2 × 50 mL) and to the filtrate was added brine and water and the layers separated. The aqueous phase was extracted with further EtOAc (2 × 50 mL), the combined organic layers washed with water (50 mL), brine (50 mL) and saturated $LiCl_{(aq)}$ (2 × 50 mL) and the organic phase was dried ($MgSO_4$) and evaporated *in vacuo*. Crude (brown solid) was purified by column chromatography on silica gel using a gradient of 0-20% EtOAc in petroleum ether to give a light yellow solid. This was recrystallised from EtOAc/petroleum ether (~1:1) to give the *title compound* as a fluffy light yellow solid (456 mg, 1.40 mmol, 37% yield). ^{1}H NMR δ_H/ppm (400 MHz, DMSO-D_6): 8.13 (1H, d, *J* 2.2 Hz, pyridyl H-6), 7.86 (1H, d, *J* 2.2 Hz, pyridyl H-4), 7.55-7.49 (2H, m, phenyl H-3, H-4), 7.42-7.38 (1H, m, phenyl H-5); ^{13}C NMR δ_C/ppm (100 MHz, DMSO-D_6): 162.7 (d, *J* 256 Hz, phenyl C-6), 158.8 (pyridyl C-2), 150.1 (pyridyl C-6), 141.6 (pyridyl C-4), 136.0 (phenyl C-2), 132.0 (d, *J* 9 Hz, phenyl C-4), 126.0 (d, *J* 3 Hz phenyl C-3), 115.3 (pyridyl C-3), 115.1 (d, *J* 21 Hz, phenyl C-5), 105.1 (phenyl C-1), 102.5 (pyridyl C-5), 94.2 (alkenyl *C*), 86.3 (alkenyl *C*); $_{max}$/cm^{-1}: 3459 (N-H), 3302 (N-H), 2163 (C≡C), 1639, 1445, 1235; HRMS *m*/*z* found: $[C_{13}H_8BrClFN_2]^+$ = 324.9426, 326.9404; $[C_{13}H_8BrClFN_2]^+$ requires 324.9543, 326.9523. NMR spectra presented in S14 Fig.

5-Bromo-2-(2-chloro-6-fluorophenyl)-1*H*-pyrrolo[2,3-*b*]pyridine (28)

To a stirring solution of 5-bromo-3-[(2-chloro-6-fluorophenyl)ethynyl]pyridin-2-amine (**27**) (453 mg, 1.39 mmol) in NMP (2 mL) under N_2 was added a suspension of potassium *tert*-butoxide (336 mg, 3.00 mmol) in NMP (2.5 mL) and the resulting dark red solution was stirred

at room temperature for 17.5 hours, then at 80 °C for 23 hours. Reaction mixture was allowed to cool to room temperature and diluted with water (10 mL), saturated $LiCl_{(aq)}$ (10 mL) and EtOAc (20 mL). Layers were separated and the aqueous phase extracted with further EtOAc (2 × 20 mL) and the combined organics washed with water (20 mL) and saturated $LiCl_{(aq)}$ (2 × 20 mL), dried ($MgSO_4$) and evaporated *in vacuo*. Crude (green gum) was purified by column chromatography on silica gel using a gradient of 0-20% EtOAc in petroleum ether, product-containing fractions were further purified using a gradient of EtOAc/petroleum ether (0-15%) to give the *title compound* as a light green solid (~85% pure by ^{1}H NMR, 119 mg, 0.32 mmol, 22% yield). ^{1}H NMR δ_H/ppm (400 MHz, $CDCl_3$): 10.62 (1H, br s, NH), 8.26 (1H, d, *J* 2.1 Hz, azaindolyl H-6), 8.10 (1H, d, *J* 2.1 Hz, azaindolyl H-4), 7.38-7.31 (2H, m, phenyl H-3, H-4), 7.19-7.14 (1H, m, phenyl H-5), 6.82 (1H, app t, *J* 1.8 Hz, azaindolyl H-3); ^{13}C NMR δ_C/ppm (100 MHz, $CDCl_3$): 160.6 (d, *J* 250 Hz, phenyl C-6), 146.9 (azaindolyl C-7a), 143.0 (azaindolyl C-6), 134.2 (d, *J* 4 Hz, azaindolyl C-2), 131.1 (azaindolyl C-4), 130.4 (d, *J* 10 Hz, phenyl C-4), 130.0 (phenyl C-2), 126.5 (d, *J* 3 Hz, phenyl C-3), 123.4 (azaindolyl C-3a), 122.0 (phenyl C-1), 114.9 (d, *J* 23 Hz, phenyl C-5), 111.9 (azaindolyl C-5), 103.7 (azaindolyl C-3); $_{max}$/cm^{-1}: 3425, 3131 (N-H), 1445, 1282, 1265, 1170; HRMS *m/z* found: $[C_{13}H_8BrClFN_2]^+$ = 324.9554. 326.9511; $[C_{13}H_8BrClFN_2]^+$ requires 324.9543, 326.9523. NMR spectra presented in S15 Fig.

5-Bromo-2,4-dimethoxypyridine (29)

To a solution of 2,4-dimethoxypyridine (835 mg, 6.01 mmol) in dry MeCN (10 mL) under N_2 was added NBS (1.176 g, 6.64 mmol) and the yellow solution heated to 75 °C and stirred for 5 hours in the dark. Solution was allowed to cool to room temperature and the solvent evaporated *in vacuo*. Residue was partitioned between EtOAc (10 mL) and water (10 mL) and the layers separated. The organic phase was washed with water (10 mL) and brine (10 mL), dried ($MgSO_4$) and evaporated *in vacuo*. Crude (yellow oil) was purified by column chromatography on silica gel using a gradient of EtOAc/petroleum ether (0-20%) to give the *title compound* as a fluffy white solid (773 mg, 3.58 mmol, 60% yield). ^{1}H NMR δ_H/ppm (400 MHz, $CDCl_3$): 8.07 (1H, s, pyridyl H-2), 6.19 (1H, s, pyridyl H-3), 3.87 (3H, s, OC*H*$_3$), 3.86 (3H, s, OC*H*$_3$); ^{13}C NMR δ_C/ppm (100 MHz, $CDCl_3$): 165.1, 163.4 (pyridyl C-2, C-4), 148.2 (pyridyl C-6), 103.0 (pyridyl C-5), 93.8 (pyridyl C-3), 56.0 (OCH_3), 53.8 (OCH_3); $_{max}$/cm^{-1}: 1586, 1556, 1460, 1366, 1209, 1163, 1066; Compound does not ionise to give correct product mass on HRMS. NMR spectra presented in S16 Fig.

(4,6-Dimethoxypyridin-3-yl)boronic acid (30)

A solution of 5-bromo-2,4-dimethoxypyridine (**29**) (299 mg, 1.38 mmol) in dry THF (5 mL) was cooled to -78 °C in a dry ice/acetone bath under N_2. *n*-butyllithium (1.6 M in hexanes, 1.5 mL, 2.4 mmol) was added dropwise and the resulting light yellow suspension stirred at -78 °C for 1 hour. Triisopropylborate (400 µL, 1.74 mmol) was added and the reaction mixture allowed to warm to room temperature and stirred for 16 hours. Saturated $NH_4Cl_{(aq)}$ (5 mL) and water (5 mL) were added to quench, followed by EtOAc (10 mL) and the layers separated. The aqueous phase was extracted with further EtOAc (2 × 10 mL) and DCM (2 × 10 mL) and the combined organics dried ($MgSO_4$) and evaporated *in vacuo*. Crude (light yellow waxy solid) was triturated with toluene (2 × 1 mL) and dried *in vacuo* to give the *title compound* as a white solid (117 mg, 0.64 mmol, 46% yield). ^{1}H NMR δ_H/ppm (400 MHz, $CDCl_3$): 8.43 (1H, s, pyridyl

H-2), 6.20 (1H, s, pyridyl H-5), 5.39 (2H, br s, boronic acid O-H), 3.97 (3H, s, OCH_3), 3.91 (3H, s, OCH_3); ^{13}C NMR δ_C/ppm (100 MHz, $CDCl_3$): 172.1 (pyridyl C-6), 168.2 (pyridyl C-4), 155.8 (pyridyl C-2), 91.8 (pyridyl C-5), 55.5 (OCH_3), 53.8 (OCH_3) (Note: A ^{13}C signal for C-3 was not present, this is frequently observed in arylboronic acids); $_{max}$/cm^{-1}: 3567 (O-H), 3183 (O-H), 1605, 1398, 1328 (B-O), 1312 (B-O), 1205, 1147, 1043; HRMS *m*/*z* found: $[C_7H_{11}BNO_4]^+$ = 184.0236; $[C_7H_{11}BNO_4]^+$ requires 184.0781. NMR spectra presented in S17 Fig.

2-(2-Chloro-6-fluorophenyl)-5-(4,6-dimethoxypyridin-3-yl)-1*H*-pyrrolo[2,3-*b*]pyridine (12)
A solution of 5-bromo-2-(2-chloro-6-fluorophenyl)-1*H*-pyrrolo[2,3-*b*]pyridine (**28**) (49 mg, 0.15 mmol), 4,6-dimethoxypyridin-3-yl)boronic acid (**30**) (39 mg, 0.22 mmol) and K_2CO_3 (64 mg, 0.46 mmol) in 1,4-dioxane (2 mL) and water (1 mL) was degassed with N_2 for 30 minutes, after which Pd(dppf)Cl_2.CH_2Cl_2 (7 mg, 0.0083 mmol) was added and the mixture heated to 70 °C and stirred for 2.5 hours. The reaction mixture was allowed to cool to room temperature, filtered through celite and the celite washed with EtOAc (2 × 10 mL). The filtrate was diluted with water (10 mL), the layers separated and the aqueous extracted with further EtOAc (2 × 10 mL). Combined organics were washed with water (20 mL), brine (20 mL), dried ($MgSO_4$) and evaporated *in vacuo*. Crude (yellow gum) was purified by column chromatography on silica gel using a gradient of EtOAc/petroleum ether (0-50%), then co-eluted fractions further purified using a gradient of EtOAc/petroleum ether (0-40%) to give the *title compound* as an off-white solid (21 mg, 0.055 mmol, 37% yield). ^{1}H NMR δ_H/ppm (400 MHz, $CDCl_3$): 10.89 (1H, br s, NH), 8.32 (1H, d, *J* 1.7 Hz, azaindolyl H-6), 8.06 (1H, d, *J* 1.7 Hz, azaindolyl H-4), 8.03 (1H, s, pyridyl H-2), 7.37-7.29 (2H, m, phenyl H-3, H-4), 7.16 (1H, app t, phenyl H-5), 6.87 (1H, s, azaindolyl H-3), 6.34 (1H, s, pyridyl H-5) 3.99 (3H, s, OCH_3), 3.85 (3H, s, OCH_3; ^{13}C NMR δ_C/ppm (100 MHz, $CDCl_3$): 165.5, 165.1 (pyridyl C-4, C-6), 160.7 (d, *J* 251 Hz, phenyl C-6), 147.9 (azaindolyl C-7a), 147.2 (pyridyl C-2), 144.4 (azaindolyl C-6), 134.3 (d, *J* 4 Hz, azaindolyl C-2), 130.0 (d, *J* 10 Hz, phenyl C-4), 129.8 (azaindolyl C-4), 128.9 (phenyl C-2), 126.3 (d, *J* 3 Hz, phenyl C-3), 123.5 (azaindolyl C-5), 120.5 (d, *J* 17 Hz, phenyl C-1), 120.1, 119.7 (azaindolyl C-3a, pyridyl C-3), 114.8 (d, *J* 23 Hz, phenyl C-5), 104.2 (d, *J* 3 Hz, azaindolyl C-3), 92.4 (pyridyl C-5), 55.5 (OCH_3), 53.6 (OCH_3); $_{max}$/cm^{-1}: 3132 (N-H), 1604, 1565, 1449, 1368, 1203, 1156, 1031; HRMS *m*/*z* found: $[C_{20}H_{16}ClFN_3O_2]^+$ = 384.0904; $[C_{20}H_{16}ClFN_3O_2]^+$ requires 384.0915. NMR spectra presented in S18 Fig.
